# Recyclable iron(ii) caffeine-derived ionic salt catalyst in the Diels–Alder reaction of cyclopentadiene and α,β-unsaturated *N*-acyl-oxazolidinones in dimethyl carbonate†

**DOI:** 10.1039/c9ra04098f

**Published:** 2019-07-15

**Authors:** Di Meng, Dazhi Li, Thierry Ollevier

**Affiliations:** Département de chimie, Université Laval 1045 Avenue de la Médecine Québec QC Canada G1V 0A6 thierry.ollevier@chm.ulaval.ca

## Abstract

Iron(ii) triflate was used in combination with caffeine-derived salts as recyclable catalysts for the Diels–Alder reaction run in dimethyl carbonate (DMC) as a green solvent. The catalyst was prepared as an ionic salt from a xanthinium salt and Fe(OTf)_2_. Various substrates including α,β-unsaturated carbonyl and *N*-acyloxazolidinone derivatives were reacted with cyclopentadiene using this recyclable catalyst. The use of a low catalyst loading (1 mol%) afforded high yields (up to 99%) of the corresponding cycloadducts. The recycling and the efficiency of the catalyst were demonstrated for several runs.

## Abstract 2

Iron(ii) triflate was used in combination with caffeine-derived salts as recyclable catalysts for the Diels–Alder reaction run in dimethyl carbonate (DMC) as a green solvent.

## Graphical abstract

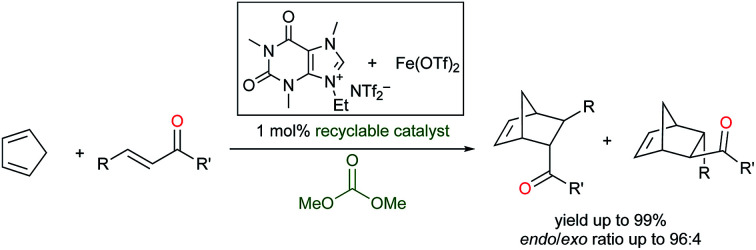

## Introduction

The Diels–Alder reaction is among the most powerful C–C bond forming transformations in synthetic chemistry.^1^ α,β-Dicarbonyl derivatives have been used as dienophiles in the Diels–Alder reaction^2^ ever since the introduction of *N*-acyloxazolinones as dienophiles 30 years ago by Evans.^3^ The reaction between α,β-unsaturated oxazolidinones and cyclopentadiene became a benchmark reaction^4^ to evaluate the catalytic activity of various metal Lewis acid catalysts, such as Mg,^5^ Cu,^6^ Sc,^7^ Ti,^8^ Ln,^2^ Ni,^9^ Pd,^10^ Fe,^11^ and Cr.^12^ Much progress was made through the development of more efficient Lewis acids and ligands.^1–12^ However, the use of large quantities of some of these catalysts to mediate this transformation, in addition to their limiting prices, render them difficult to use, thus creating a need for additional recyclable catalysts.^13^ Various approaches include heterogeneous catalysis,^14^ or replacing the reaction solvent by ionic liquids (ILs), which were originally used toward improved stereoselectivity.^14,15^ Ionic liquids have received considerable attention as new powerful reaction media and emerged as a potential alternative to conventional organic solvents.^16^ Furthermore, the combination of ionic liquids or ionic salts with transition metals has been reported as a promising area, and their scope of applicability is extending.^17^ An immobilized catalytic system of [bmim][FeCl_4_] was developed for aryl Grignard cross-coupling *via* a liquid–liquid biphasic process, in which FeCl_3_ was trapped in the ionic liquid.^18^ A similar system involving a Bi(OTf)_3_-trapped caffeine-derived salt (xanthinium salt) in a Diels–Alder reaction, in which the combined xanthinium–Bi^III^ mixture of salts was recycled without loss of efficiency over several runs.^19^ However, one drawback of these methods was the use of dichloromethane as a solvent. In fact, the most commonly used solvents in Diels–Alder reactions are, among others, dichloromethane, chloroform, toluene, diethyl ether, and water. Hence, in order to develop a Diels–Alder reaction run in greener conditions, we were interested in seeking appropriate environmentally-benign solvents. Although ILs have many advantages, their high prices and waste disposal during large scale applications limit their widespread use, not to mention their adverse environmental impact in their life cycle assessment (LCA).^20^ Beside ILs, other ecofriendly solvents have also been studied.^21^ Dimethyl carbonate (DMC), which has been assessed as a green alternative to replace easily-flammable organic compounds (VOC) as solvents,^22^ appeared promising to use. It is environmentally benign and scores low on LCA scales.^23^ In the context of our research on iron catalysis and ionic liquids,^24^ a combination of a caffeine derivative, an iron salt, and dimethyl carbonate is disclosed herein.

Iron has attracted considerable attention on its eco-friendliness, natural abundance, inexpensive price, and its promising applications in organic synthesis.^25^ Fe(OTf)_2_ was already used with an ionic liquid ethylmethylimidazolium bis-triflimide in an aziridination reaction.^26^ Also, Fe^II^/Fe^III^-derived catalysts have been used in the Diels–Alder reaction, including asymmetric versions.^4*a*,27^ Moreover, many other examples of Fe^II^/Fe^III^-catalyzed Diels–Alder reactions were reported in homogeneous and heterogeneous catalysis.^28^

**Scheme 1:**
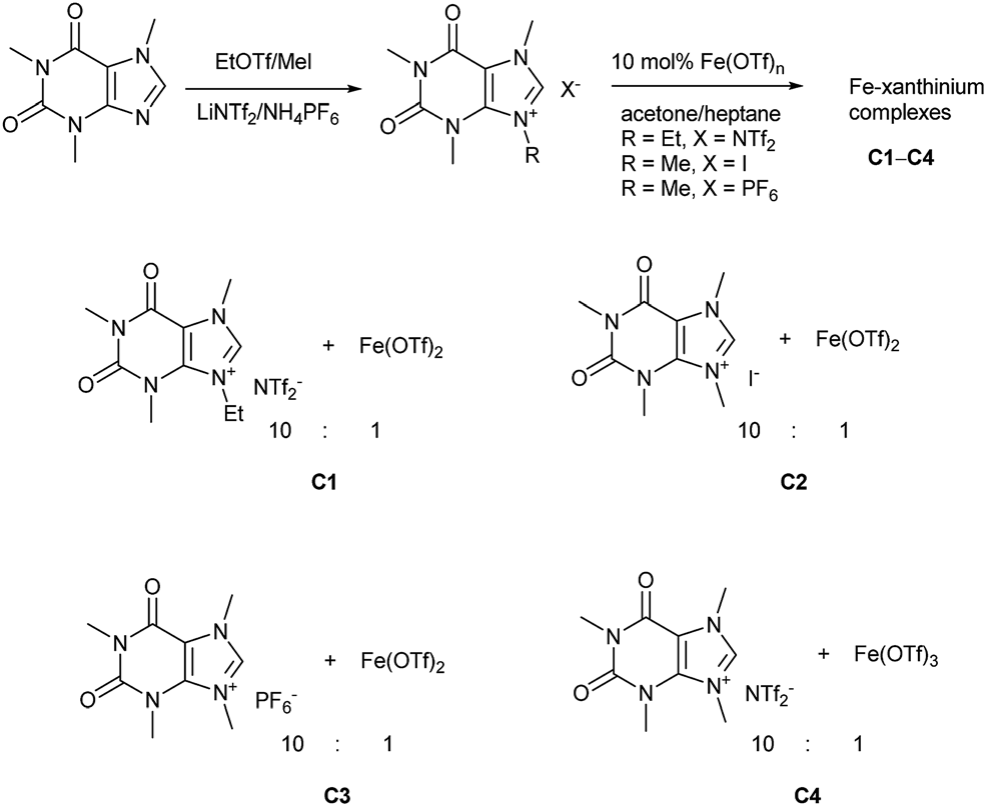
Synthetic routes of chiral dihydroquinoxalinones.

Caffeine, as one of the methylxanthines, is a green, natural, abundant, and biodegradable compound that can be further alkylated into xanthinium salts.^29^ These xanthinium salts have been used for the preparation of NHC-metal complexes in medicinal and organometallic chemistry.^30^ As already mentioned, a caffeine-derived ethylxanthinium salt was already used as an ionic solid in a 10 : 1 ratio to Bi(OTf)_3_·4H_2_O and recycled as a combined ionic salt.^18,19^ The recycling of this catalyst was easily performed after its precipitation from heptane; however, the reaction was run in CH_2_Cl_2_ and only a few dienophiles were disclosed.^18,19^ Alkylated caffeines, *N*-methyl- and *N*-ethyl-substituted xanthinium salts were obtained with NTf_2_^−^, I^−^, and PF_6_^−^ anions (Scheme 1).^18,19,30*b*–*g*^ Next, the xanthiniums were mixed with Fe(OTf)_2_ or Fe(OTf)_3_ in acetone (Scheme 1). The ratio of the xanthiniums to Fe^II^/Fe^III^ salts was examined in a range of 2 : 1 to 10 : 1. For ratios 7 : 1 to 10 : 1, the iron salt was completely solubilized. A 10 : 1 ratio was used to ensure that there was no loss of the catalyst during the recycling process. Acetone and heptane were used to prepare catalysts C1, C2, C3, and C4. These catalysts were tested conjointly with Fe(OTf)_2_ and Fe(NTf_2_)_2_ in the Diels–Alder reaction of cyclopentadiene and 3-acryloyl-1,4-oxazolidin-2-one, chosen as the model reaction.

## Results and discussion

Initially, Fe(OTf)_2_ and catalyst C1 were studied in dichloromethane. The ratio of diene to dienophile was set to 7 : 1. The yields and stereoselectivities obtained with Fe(OTf)_2_ (Table 1, entry 1) and catalyst C1 in CH_2_Cl_2_ were similar (entry 2). The same yield and *endo*/*exo* selectivity were obtained in dimethyl carbonate at room temperature (entry 3) and even at 2 °C (melting point of DMC, entry 4). Then, an optimization study was performed by lowering the ratio of reactants from 7 : 1 to 5 : 1 (entry 5) until 2 : 1, and a quantitative yield together with a slightly higher *endo*/*exo* ratio was obtained (entry 6). This result was even better than our previous study using Fe^III^ catalyst in DMC (76% yield).^24*e*^ The other three catalysts C2, C3, and C4 were tested in the same conditions as in entry 6. Catalysts C2 and C3 led to low yields of 3a (entries 7 and 8). Using the Fe(OTf)_3_ derived catalyst C4, the yield reached 97%, whereas the *endo*/*exo* ratio decreased to 90 : 10 (entry 9). Three control experiments were consequently performed (entries 10, 11, and 12). The yield dropped to 34% without using a catalyst (entry 10). While Fe(OTf)_2_ led to a lower yield than when using catalyst C1 (entry 11). A quantitative yield was obtained when using Fe(NTf_2_)_2_ alone (entry 12). Compared to Fe(OTf)_2_, catalyst C1 was shown to be more efficient in terms of yield and stereoselectivity.

**Table 1:** Optimization of the reaction between cyclopentadiene and 3-acryloyl-1,4-oxazolidin-2-one^a^

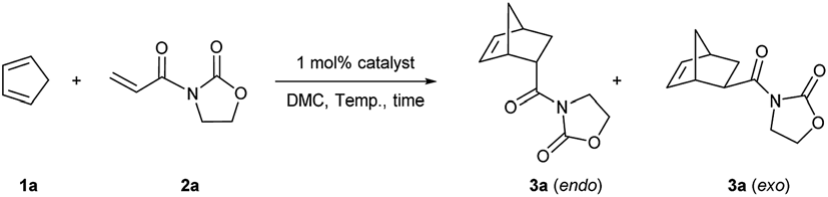						
Entry	1a/2a	Catalyst	*T* (°C)	*t* (h)	*Endo*/*exo*	Yield 3a^d^ (%)
1^b^	7 : 1	Fe(OTf)_2_	rt	13	87 : 13	89
2^b^	7 : 1	C1	rt	13	88 : 12	92
3	7 : 1	C1	rt	13	91 : 9	99
4	7 : 1	C1	2	3	91 : 9	99
5	5 : 1	C1	2	3	91 : 9	99
6	2 : 1	C1	2	3	94 : 6	99
7	2 : 1	C2	2	3	94 : 6	33
8	2 : 1	C3	2	3	90 : 10	55
9	2 : 1	C4	2	3	90 : 10	97
10	2 : 1	—	2	3	90 : 10	34
11	2 : 1	Fe(OTf)_2_	2	3	90 : 10	81
12	2 : 1	Fe(NTf_2_)_2_	2	3	93 : 7	100
13^c^	2 : 1	C1	2	3	94 : 6	100

a Conditions: 1a (1 mmol), 2a (0.5 mmol), DMC (1 mL); 3a_*endo*_/3a_*exo*_ ratio was calculated by ^1^H NMR.

b Reaction run in CH_2_Cl_2_ (1 mL).

c Reaction was run on 7.1 mmol of 2a (1 g of scale).

d Isolated yields.

Although Fe(NTf_2_)_2_ alone was as efficient as catalyst C1, it was not recyclable from the reaction media. Similarly, Fe(OTf)_2_ was also lost during work-up (entries 1 and 11). On the contrary, catalyst C1 could be recycled after the addition of heptane and filtration. Since no detectable difference was observed among the results obtained with C1 and Fe(NTf_2_)_2_, it was hypothesized that OTf^−^ and NTf_2_^−^ can readily exchange within the catalytic system (entries 2 and 12). The reaction using gram scale of 2a was tested as well, and a quantitative yield was obtained (entry 13).

To explore the scope of the reaction solvent, a few green solvents were selected and compared with CH_2_Cl_2_ and THF (Table 2). CH_2_Cl_2_ afforded the lowest *endo*/*exo* selectivity, while THF led to a moderate yield and good *endo*/*exo* selectivity (entries 1 and 2). Me-THF resulted in an even lower yield than THF, but with a high stereoselectivity (entry 3). *N*-Methyl pyrrolidinone (NMP) was found less suitable due to its high boiling point, resulting in a difficult separation from the reaction product (entry 4). Cyclopentyl methyl ether (CPME), ethyl acetate, and methyl *tert*-butyl ether (MTBE) led to good results (entries 5, 6, and 7). Of all the solvents tested, DMC was selected for its polarity close to CH_2_Cl_2_.^31^ It provided a high *endo*/*exo* ratio combined with an excellent yield (entry 8) and was thus chosen for the next part of the study.

**Table 2:** Solvent optimization for the Diels–Alder reaction of cyclopentadiene (1a) and 3-acryloyl-1,4-oxazolidin-2-one (2a)^a^

Entry	Solvent (polarity)^b^	*Endo*/*exo*	Yield 3a^c^ (%)
1	CH_2_Cl_2_ (0.309)	76 : 24	92
2	THF (0.207)	80 : 20	65
3	Me-THF (0.179)	90 : 10	50
4	NMP (0.355)	88 : 12	85
5	CPME (—)	77 : 23	100
6	EtOAc (0.228)	80 : 20	98
7	MTBE (0.124)	79 : 21	97
8	DMC (0.232)	94 : 6	99

a Conditions: 1a (1.0 mmol), 2a (0.50 mmol), C1 (1 mol%), DMC (1 mL), 2 °C, 3 h; *endo*/*exo* ratio was determined by ^1^H NMR.

b Relative polarity using water as reference (polarity = 1).^31^

c Isolated yields.

To investigate the recoverability of the catalyst, recycling tests were performed using C1. Table 3 highlights the reaction yields and the mass of recycled catalyst after each of the 5 runs of the reaction. Firstly, 1 mol% catalyst was used for five runs, and after each run, heptane was added to the reaction mixture to precipitate the catalyst. Then, after filtration through a cotton-plugged pipet, the catalyst was recollected and washed with acetone, and was finally recycled after evaporation. Using 1 mol% of C1, the yields of both the product and the recycled catalyst slightly decreased over the 5 runs.^32^ However, when the catalyst loading was increased to 2 mol%, all of the catalyst was recycled after each run, and the reaction yield was maintained at the same level. According to ^1^H and ^19^F NMR analysis, the chemical composition of the catalyst resulted unchanged (or mostly unchanged) after recovery (see ESI†).

**Table 3:** Recycling study of catalyst C1 and yields of the Diels–Alder reaction over 5 runs^a^

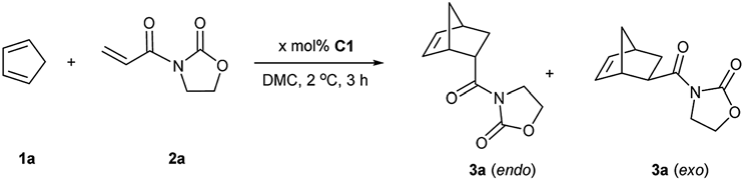						
*x* mol%		Run 1	Run 2	Run 3	Run 4	Run 5
1	Yield (%)	99	98	96	95	95
	Cat. recycl. (%)	100	99	97	95	94
2	Yield (%)	99	99	99	99	99
	Cat. recycl. (%)	100	100	100	100	100

a Conditions: **1a** (1 mmol), **2a** (0.5 mmol), **C1** (1 mol%), DMC (1 mL); isolated yields.

**Scheme 2:**
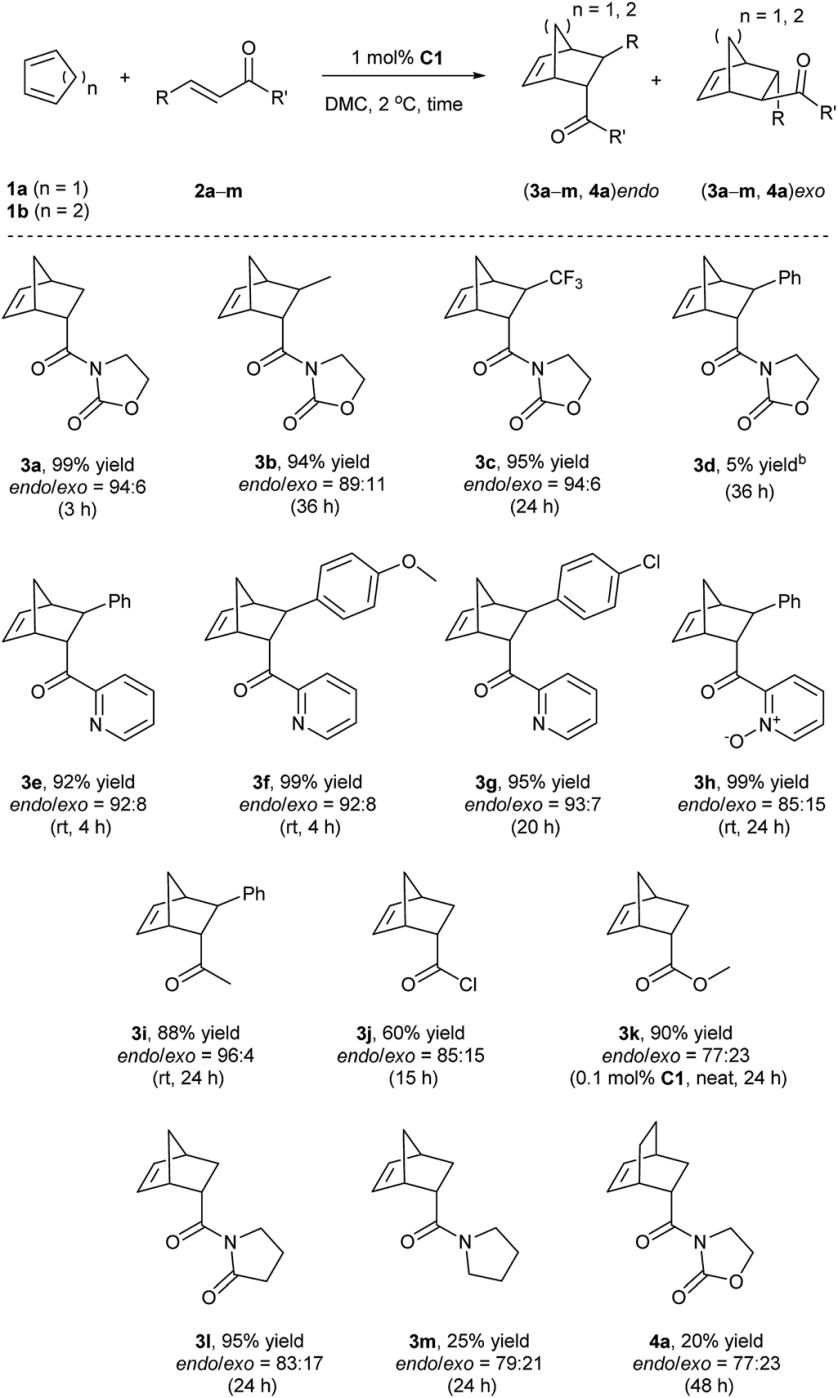
Reaction scope between cyclic dienes and various dienophiles.^*a a*^ Conditions: 1a or 1b (1 mmol), 2a–2m (0.5 mmol), C1 (1 mol%), DMC (1 mL); isolated yields unless stated otherwise; *endo*/*exo* ratio was determined by ^1^H NMR. ^*b*^ Yield was calculated by ^1^H NMR.

Finally, given the results above, the optimized conditions were applied to various dienophiles in the Diels–Alder reaction (Scheme 2). The general procedure was run as follows: cyclopentadiene was added into a solution of the catalyst and the dienophile in dimethylcarbonate. After the given time, heptane was added into the solution to precipitate the catalyst for recycling. 3-Crotonyl-oxazolidinone (2b) required a longer time than substrate acryloyl-oxazolidin-2-one (2a) but afforded a slightly higher stereoselectivity of 3b. Instead of a CH_3_ group, a β-CF_3_ to the carbonyl group led to higher *endo*/*exo* selectivity and high yield (3c). However, low solubility of cinnamoyl oxazolidinone resulted in a very low yield (3d).^33^ 2-Alkenoyl pyridines were explored as bidentate dienophiles that can chelate to the metal center through both the pyridine and the carbonyl lone pairs.^6*a*^ Here, the objective was to use them as substrates in the developed conditions (3e–h). High yields and high *endo*/*exo* ratios were obtained. The stereoselectivity decreased when using cinnamoyl pyridine *N*-oxide (2h), probably due to the weaker coordination of the oxide with iron than that with pyridine. Being similar on the cinnamoyl part, benzylidene acetone (2i) and acryloyl chloride (2j) were chosen to get a comparison with the bidentate dienophiles 2d and 2e. Benzylidene acetone showed a good reactivity with a higher *endo*/*exo* ratio (3i). Using acryloyl chloride, a moderate yield (60%, 3j) was obtained. Neat conditions were efficiently used with methyl acrylate at very low catalyst loading (0.1 mol%), and high yield (90%), and selectivity were obtained (77 : 23 *endo*/*exo*, 3k), which was even better than some obtained results using ionic liquids, but low yield was obtained for methyl propiolate.^34^ While keeping the dicarbonyl group, acryloyl 2-pyrrolidinone led to a 95% yield and a 83 : 17 *endo*/*exo* selectivity (3l). Without the second carbonyl group, the yield dropped to 25% when using acryloyl amide (2m), which revealed the importance of the oxazolidin-2-one or pyridine group to obtain a good conversion. Finally, cyclohexa-1,3-diene was selected to react with 3-acryloyl-1,4-oxazolidin-2-one, but this diene led to a slower reaction rate. A 20% yield and 77 : 23 *endo*/*exo* ratio were obtained for 4a.

**Fig. 1:**
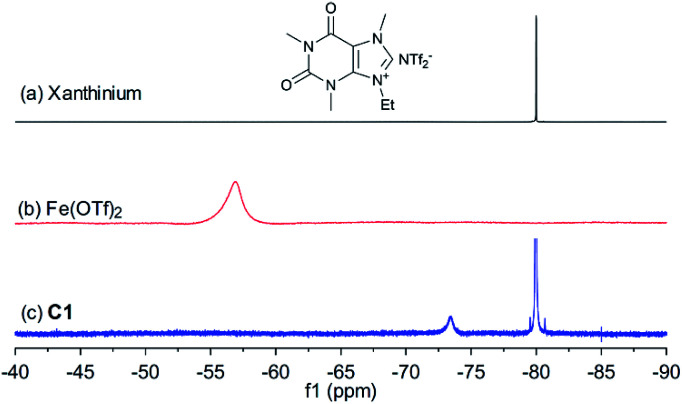
^19^F NMR spectrum of (a) xanthinium, (b) Fe(OTf)_2_ and (c) C1 (376 MHz, (CD_3_)_2_CO).

In order to shed light on the nature of the catalyst, the interaction between xanthinium and Fe(OTf)_2_ in the catalyst was studied by UV-vis, FTIR, ^1^H, ^13^C, and ^19^F NMR, and HRMS techniques (see ESI† for more details). As shown in Fig. 1, after mixing xanthinium and Fe(OTf)_2_ together, the ^19^F NMR signal of OTf^−^ shifted from −56.9 ppm to −73.4 ppm, which is comparable to the chemical shift of unbound OTf^−^ in Fe^2+^ complexes (−69.7 ppm,^35^ −78.96 ppm,^36^ and −79.59 ppm^37^). Since the OTf^−^ signal shifted to high field (*i.e.* became more electron-enriched), the counterpart (Fe^2+^) was considered more electron-deficient, thus more Lewis acidic for the activation of dienophiles. The increase of Lewis acidity of Fe(ii) in C1 thus led to higher yield (Table 1, entry 6 *vs.* entry 11). On the other hand, the NTf_2_^−^ is a non-coordinating anion, so the ^19^F signal of NTf_2_^−^ (−80.0 ppm) did not considerably change. According to the changes observed by FTIR, ^19^F NMR, and HRMS, the interaction between xanthinium and Fe(OTf)_2_ has been highlighted and provides evidence for anion metathesis.^37^ DFT calculations (B3LYP/6-31G/LANL2DZ level in gas phase) were run to shed light on the changes of Gibbs free energy and Mulliken charges of Fe(ii) after the ion exchanges. According to the calculations, the anion exchange in C1 is favored (Δ*G* = −22.2 kcal mol^−1^), which is supported by the ^19^F NMR change and HRMS, allowing a qualitative evaluation of C1–C4 (see ESI† for more details).

## Conclusions

In conclusion, a recyclable ionic salt/iron triflate catalyst was prepared from a xanthinium salt and iron(ii) triflate. This green catalytic system using a caffeine-derived xanthinium–Fe(OTf)_2_ complex for the Diels–Alder reaction run in DMC was developed for a large scope of substrates. The xanthinium salt as a solid provides a new way of catalyst immobilization in comparison to ionic liquids. Several green solvents were examined and the recycling of the catalyst was demonstrated for several runs. The use of a caffeine derivative, an iron salt, and dimethyl carbonate represents a major advancement from a green chemistry point of view. Work is in progress on further applications of ionic salt catalysts and will be reported in due course.

## Experimental

### General information

All materials are commercially available and were used as received without further purification. Thin-layer chromatography (TLC) was performed on commercial silica gel plates (250 μm, Silicycle F254) and compounds were visualized using UV light or KMnO_4_. The compounds were purified on silica gel column (200–300 mesh) unless stated otherwise. IR spectra were measured on a Bomem Michelson 100 Series FTIR spectrometer. ^1^H, ^13^C, and ^19^F NMR spectra were recorded on a Bruker AC 300 MHz, Varian Inova 400 MHz or Agilent Technologies DD2 500 MHz spectrometers. Chemical shifts are given in ppm and residual solvent peaks were used as reference. The coupling constants were reported in hertz. High-resolution mass spectra (HRMS) were recorded on a LC/MS-TOF Agilent 6210 mass spectrometer (electrospray ionization). Xanthiniums were synthesized according to published procedures.^19,30*a*,38^

### Preparation of catalysts C1–C4

The 1,3,7-trimethyl-9-ethylxanthinium bis(trifluoromethanesulfonyl)amide (1.26 g, 2.5 mmol) and anhydrous Fe(OTf)_2_ (89 mg, 0.25 mmol) were added to a 25 mL oven-dried flask. Acetone (7 mL) was then added into the flask to dissolve the salts, then heptane (15 mL) was added. A brown solution formed at the bottom of the flask. Then, the solvent was slowly evaporated at reduced pressure, and the flask was put under high vacuum overnight. Catalyst C1 was obtained in quantitative yield. Catalysts C2–C4 were prepared according to the same procedure. Characterization data for each catalyst is shown below.

#### Catalyst C1

^1^H NMR (400 MHz, (CD_3_)_2_CO) *δ* 9.29 (s, 1H, N–C*H*=N), 4.87 (q, *J* = 7.2 Hz, 2H, –C*H*_2_CH_3_), 4.26 (s, 3H, –C*H*_3_), 3.91 (s, 3H, –C*H*_3_), 3.35 (s, 3H, –C*H*_3_), 1.72 (t, *J* = 7.2 Hz, 3H, –CH_2_C*H*_3_); ^1^H NMR (400 MHz, (CD_3_)_2_SO) *δ* 9.33 (s, 1H), 4.53 (q, *J* = 7.2 Hz, 2H), 4.03 (s, 3H), 3.69 (s, 3H), 3.25 (s, 3H), 1.48 (t, *J* = 7.2 Hz, 3H). ^13^C NMR (101 MHz, (CD_3_)_2_SO) *δ* 153.75 (C–*C*=O–N), 150.77 (N–*C*=O–N), 139.28 (Ar), 139.14 (N–*C*H=N), 119.87 (q, *J*_CF_ = 321.7 Hz, NTf_2_^−^), 108.30 (Ar), 45.43 (–*C*H_2_CH_3_), 36.10 (–*C*H_3_), 32.02 (–*C*H_3_), 28.78 (–*C*H_3_), 15.54 (–CH_2_*C*H_3_) (signal of OTf^−^ was not observed). ^19^F NMR (282 MHz, (CD_3_)_2_CO) *δ* −73.40 (s, OTf^−^), −79.97 (s, NTf_2_^−^); ^19^F NMR (376 MHz, (CD_3_)_2_SO) *δ* −77.84 (s, OTf^−^), −78.78 (s, NTf_2_^−^). IR (ZnSe): 3429, 3164, 3100, 2960, 1721, 1670, 1580, 1545, 1450, 1347, 1321, 1176, 1048, 853, 739 cm^−1^.

#### Catalyst C2

^1^H NMR (400 MHz, (CD_3_)_2_CO) *δ* 9.48 (s, 1H), 4.44 (s, 3H), 4.25 (s, 3H), 3.93 (s, 3H), 3.34 (s, 3H); ^1^H NMR (400 MHz, (CD_3_)_2_SO) *δ* 9.27 (s, 1H), 4.12 (s, 3H), 4.03 (s, 3H), 3.71 (s, 3H), 3.24 (s, 3H). ^13^C NMR (101 MHz, (CD_3_)_2_SO) *δ* 153.71 (C–*C*=O–N), 150.59 (N–*C*=O–N), 140.01 (Ar), 139.68 (N–*C*H=N), 108.16 (Ar), 37.28 (–*C*H_3_), 36.04 (–*C*H_3_), 31.77 (–*C*H_3_), 28.82 (–*C*H_3_) (signal of OTf^−^ was not observed). ^19^F NMR (376 MHz, (CD_3_)_2_CO) *δ* −79.06 (s, OTf^−^). IR (ZnSe): 3489, 3422, 3095, 3047, 1716, 1670, 1581, 1543, 1459, 1031, 1283, 1258, 1167, 1029, 1001, 876, 790, 776, 736 cm^−1^.

#### Catalyst C3

^1^H NMR (400 MHz, (CD_3_)_2_CO) *δ* 9.15 (s, 1H), 4.44 (s, 3H), 4.25 (s, 3H), 3.93 (s, 3H), 3.35 (s, 3H). ^13^C NMR (75 MHz, (CD_3_)_2_CO) *δ* 153.49, 139.96, 139.40, 108.52, 36.97, 35.61, 31.10, 27.92 (one of carbonyls and OTf^−^ was not observed). ^19^F NMR (376 MHz, (CD_3_)_2_CO) *δ* −72.77 (d, *J*_PF_ = 707.7 Hz, PF_6_^−^) (signal of OTf^−^ was not observed). IR (ZnSe): 3392, 3181, 3122, 1717, 1661, 1581, 1547, 1461, 1348, 1182, 1105, 1054, 1031, 812, 743 cm^−1^.

#### Catalyst C4

^1^H NMR (300 MHz, (CD_3_)_2_SO) *δ* 9.23 (s, 1H), 4.10 (s, 3H), 4.01 (s, 3H), 3.70 (s, 3H), 3.23 (s, 3H). ^19^F NMR (282 MHz, (CD_3_)_2_SO) *δ* −77.64 (s, NTf_2_^−^) (signal of OTf^−^ was not observed). IR (ZnSe): 3557, 3171, 3100, 2363, 1844, 1723, 1681, 1583, 1548, 1464, 1352, 1189, 1137, 1054, 746 cm^−1^.

### General procedure for Diels–Alder reaction in DMC

To an oven-dried vial, were added catalyst C1 (27.5 mg, 0.005 mmol) and dienophile 3-acryloyl-1,3-oxazolin-2-one (70.5 mg, 0.5 mmol), then DMC (1 mL) was injected. The solution was stirred for 10 minutes. After that, the vial was placed in an ice water bath at 2 °C. Then the pre-distilled cyclopentadiene (1 mmol, 66 mg) was injected into the vial. The solution was stirred for 3 hours. After the reaction was complete, heptane (5 mL) was added into the solution to precipitate the catalyst (other solvents, such as hexane, petroleum ether and Et_2_O, could also be used for recycling of catalyst). The precipitate of catalyst was recycled (27.5 mg) by filtration. The filtrate was concentrated, and the crude product was purified by silica gel column chromatography using hexanes/EtOAc as the eluent to obtain the product (103 mg, 99% yield). For other substrates, 0.25 mmol of dienophiles and 0.5 mmol of diene were used with 0.0025 mmol (14 mg) of the catalyst, and the crude products were purified by silica gel column chromatography using hexanes/EtOAc as the eluent unless stated otherwise.

### General procedure for Diels–Alder reaction under neat conditions

For liquid dienophiles, the reactions were carried out in neat conditions. Typically, to an oven-dried vial, was added catalyst C1 (27.5 mg, 0.005 mmol). The reaction vial was then cooled at 2–3 °C using a cryostat. Methyl acrylate (430 mg, 5 mmol) and distilled cyclopentadiene (396 mg, 6 mmol, 1.2 equiv.) were then injected into the vial. The reaction mixture was stirred for 1 day. Then, heptane (5 mL) was added into the vial to precipitate the catalyst, and the crude product was obtained through filtration of cotton-plugged pipet. The filtrate was evaporated and the product was dried under high vacuum for 5–10 minutes. Pure product was obtained without further purification in 90% yield (677 mg). The precipitated catalyst was dissolved by acetone, and filtered through cotton-plugged pipet to be recycled after evaporation of acetone. All the mass of the catalyst (27.5 mg) was recycled. Characterization data for each product is shown below.

#### 3-(Bicyclo[2.2.1]hept-5-ene-2-carbonyl)oxazolidin-2-one (3a)^27*b*^

The product was obtained as a colorless semi-solid (103 mg, 0.495 mmol, 99%). ^1^H NMR (500 MHz, CDCl_3_) *endo*: *δ* 6.23 (dd, *J* = 5.3, 2.9 Hz, 1H), 5.86 (dd, *J* = 5.4, 2.6 Hz, 1H), 4.43–4.35 (m, 2H), 3.99–3.89 (m, 3H), 3.29 (br, 1H), 2.93 (br s, 1H), 1.94 (m, 1H), 1.49–1.37 (m, 3H); *exo* (specific protons): *δ* 6.16 (s, 2H), 4.05–4.00 (m, 3H), 3.27–3.24 (m, 1H), 3.00 (s, 1H), 1.51 (m, 1H), 1.35 (m, 1H). ^13^C NMR (126 MHz, CDCl_3_), *endo*: *δ* 174.7, 153.4, 138.1, 131.6, 62.0, 50.2, 46.3, 43.1, 42.9, 42.9, 29.5; *exo*: *δ* 176.1, 153.3, 138.2, 135.9, 61.9, 46.8, 46.1, 43.1, 43.0, 41.9, 30.4. IR (NaCl): 2975, 1775, 1696, 1386, 1225, 1040, 1005, 761, 705 cm^−1^.

#### 3-(3-Methylbicyclo[2.2.1]hept-5-ene-2-carbonyl)oxazolidin-2-one (3b)^27*b*^

The product was obtained as a white solid (104.0 mg, 0.470 mmol, 94%). ^1^H NMR (400 MHz, CDCl_3_) *endo*: *δ* 6.36 (dd, *J* = 5.6, 3.1 Hz, 1H), 5.76 (dd, *J* = 5.7, 2.8 Hz, 1H), 4.39 (td, *J* = 8.0, 3.1 Hz, 2H), 4.03–3.88 (m, 2H), 3.51 (dd, *J* = 4.3, 3.5 Hz, 1H), 3.26 (br s, 1H), 2.51 (br s, 1H), 2.12–2.02 (m, 1H), 1.69 (d, *J* = 8.7 Hz, 1H), 1.44 (dq, *J* = 8.6, 1.6 Hz, 1H), 1.11 (d, *J* = 7.1 Hz, 3H); *exo* (significant peaks): *δ* 6.30 (dd, *J* = 5.6, 3.1 Hz, 1H), 6.14 (dd, *J* = 5.6, 2.9 Hz, 1H). ^13^C NMR (126 MHz, CDCl_3_) *endo*: *δ* 174.4, 153.5, 139.7, 130.9, 61.9, 51.3, 49.5, 47.5, 47.1, 43.0, 36.4, 20.4; *exo*: *δ* 175.54, 153.44, 136.87, 135.53, 61.82, 50.64, 49.52, 47.50, 46.66, 43.07, 37.35, 18.84. IR (NaCl): 2963, 1776, 1696, 1386, 1229, 1100, 770, 735, 705 cm^−1^.

#### 3-(3-(Trifluoromethyl)bicyclo[2.2.1]hept-5-ene-2-carbonyl)oxazo-lidin-2-one (3c)

The product was obtained as a white solid (65 mg, 0.236 mmol, 95%). Mp 79–80 °C. ^1^H NMR (500 MHz, CDCl_3_) *δ* 6.41–6.35 (m, 1H for *endo* ((6.38, dd, *J* = 5.0, 3.6 Hz)), 1H for *exo*), 6.14 (m, 1H), 4.47–4.39 (m, 2H), 4.14–3.99 (m, 2H), 3.52 (m, 1H), 3.44 (d, *J* = 5.6 Hz, 1H), 3.15 (br, 1H), 3.04 (br, 1H), 1.58 (d, *J* = 8.9 Hz, 1H), 1.43 (d, *J* = 8.9 Hz, 1H); *exo*: 5.90 (dd, *J* = 5.6, 2.8 Hz, 1H) 3.93 (m, 1H), 3.10 (d, *J* = 1.2 Hz, 1H), 2.76 (qdd, *J* = 10.3, 5.5, 1.3 Hz, 1H) 1.82 (dd, *J* = 9.0, 1H), 1.51 (d, *J* = 9.0 Hz, 1H). ^13^C NMR (126 MHz, CDCl_3_) *endo*: *δ* 172.4, 153.3, 137.2, 134.7, 127.1 (q, *J* = 277.5 Hz), 62.1, 49.3, 46.2 (d, *J* = 1.0 Hz), 46.00 (d, *J* = 1.3 Hz), 45.3 (q, *J* = 26.9 Hz), 43.4 (q, *J* = 1.7 Hz), 42.9; *exo*: *δ* 171.74, 153.3, 138.7, 133.2, 127.4 (q, *J* = 278.3 Hz), 62.1, 46.5, 48.3 (d, *J* = 1.3 Hz), 44.8 (d, *J* = 1.3 Hz), 45.4 (q, *J* = 27.0 Hz), 44.0 (d, *J* = 1.7 Hz), 42.9. IR (NaCl): 2990, 1779, 1696, 1362, 1284, 1125, 676 cm^−1^. HRMS (ESI): *m*/*z* calcd for C_12_H_12_F_3_NNaO_3_ [M + Na]^+^ 298.0662, found 298.0654.

#### (3-Phenylbicyclo[2.2.1]hept-5-en-2-yl)(pyridin-2-yl)methanone (3e)^39^

The product was obtained as a white solid (63 mg, 0.229 mmol, 92%). Mp 45–46 °C. ^1^H NMR (400 MHz, CDCl_3_) *endo*: *δ* 8.69 (dd, *J* = 4.7, 0.6 Hz, 1H), 8.03 (d, *J* = 7.8 Hz, 1H), 7.82 (td, *J* = 7.7, 1.7 Hz, 1H), 7.45 (ddd, *J* = 7.5, 4.8, 1.1 Hz, 1H), 7.37–7.28 (m, 4H), 7.16 (m, 1H), 6.52 (dd, *J* = 5.5, 3.2 Hz, 1H), 5.86 (dd, *J* = 5.6, 2.7 Hz, 1H), 4.57 (dd, *J* = 5.1, 3.5 Hz, 1H), 3.58 (br s, 1H), 3.49 (d, *J* = 4.5 Hz, 1H), 3.12 (d, *J* = 1.2 Hz, 1H), 2.10 (d, *J* = 8.4 Hz, 1H), 1.64 (dd, *J* = 8.4, 1.6 Hz, 1H); *exo* (specific peaks): *δ* 8.67 (dd, *J* = 4.7, 0.7 Hz, 1H), 8.09 (d, *J* = 7.8 Hz, 1H), 7.27–7.23 (m, 4H), 6.55–6.54 (m, 1H), 6.13 (dd, *J* = 5.5, 2.8 Hz, 1H), 4.24 (dd, *J* = 5.3, 0.9 Hz, 1H), 4.06 (dd, *J* = 5.2, 3.5 Hz, 1H), 3.23 (s, 1H), 3.13 (s, 1H), 1.90 (d, *J* = 8.5 Hz, 1H), 1.50 (dd, *J* = 8.5, 1.4 Hz, 1H). ^13^C NMR (126 MHz, CDCl_3_) *endo*: *δ* 201.09, 153.57, 148.89, 144.64, 139.44, 136.85, 132.88, 128.40, 127.67, 126.92, 125.85, 122.20, 54.28, 49.37, 48.78, 48.26, 45.59; *exo*: *δ* 202.20, 153.40, 148.97, 143.72, 137.05, 136.76, 136.19, 128.16, 127.91, 126.91, 125.90, 122.40, 52.10, 49.35, 48.99, 47.00, 46.92. IR (NaCl): 2974, 1690, 1600, 1568, 1497, 1332, 1272, 1019, 995, 740 cm^−1^.

#### (3-(4-Methoxyphenyl)bicyclo[2.2.1]hept-5-en-2-yl)(pyridin-2-yl)methanone (3f)^39^

The product was obtained as a colorless oil (76 mg, 0.249 mmol, 99%). ^1^H NMR (400 MHz, CDCl_3_) *endo*: *δ* 8.70–8.67 (m, 1H), 8.01 (d, *J* = 7.9 Hz, 1H), 7.81 (td, *J* = 7.7, 1.7 Hz, 1H), 7.44 (m, 1H), 7.27–7.23 (m, 2H), 6.84 (dd, *J* = 8.5, 1.6 Hz, 2H), 6.50 (m, 1H), 5.82 (m, 1H), 4.50 (m, 1H), 3.78 (d, *J* = 1.8 Hz, 3H), 3.54 (br s, 1H), 3.40 (d, *J* = 5.0 Hz, 1H), 3.04 (br, 1H), 2.07 (d, *J* = 8.4 Hz, 1H), 1.61 (d, *J* = 8.4 Hz, 1H); *exo*: (specific protons): *δ* 8.65 (m, 1H), 8.07 (d, *J* = 7.9 Hz, 1H), 7.14 (d, *J* = 7.3 Hz, 1H), 6.78 (dd, *J* = 8.5, 1.6 Hz, 2H), 6.52 (m, 1H), 6.12 (m, 1H), 4.16 (d, *J* = 5.3 Hz, 1H), 3.96 (m, 1H), 3.76 (d, *J* = 1.8 Hz, 3H), 3.16 (s, 1H), 3.10 (s, 1H), 1.86 (d, *J* = 8.5 Hz, 1H), 1.47 (d, *J* = 8.5 Hz, 1H). ^13^C NMR (126 MHz, CDCl_3_) *endo*: *δ* 201.2, 157.7, 153.56, 148.9, 139.4, 136.8, 136.6, 132.8, 128.6, 126.90, 122.2, 113.8, 55.3, 54.3, 49.7, 48.7, 48.2, 44.9; *exo*: *δ* 202.3, 157.81, 153.4, 149.0, 137.0, 136.8, 136.2, 135.8, 129.0, 126.9, 122.4, 113.3, 55.2, 52.3, 49.30, 49.1, 47.00, 46.2. IR (NaCl): 2970, 1689, 1610, 1582, 1569, 1512, 1249, 1036, 678, 603 cm^−1^.

#### (3-(4-Chlorophenyl)bicyclo[2.2.1]hept-5-en-2-yl)(pyridin-2-yl)methanone (3g)^39^

The product was obtained as a colorless oil (57.9 mg, 0.238 mmol, 96%). ^1^H NMR (400 MHz, CDCl_3_) *endo*: *δ* 8.68–8.66 (m, 1H), 8.01 (dt, *J* = 7.9, 0.9 Hz, 1H), 7.81 (m, 1H), 7.45 (m, 1H), 7.24–7.12 (m, 4H), 6.48 (dd, *J* = 5.6, 3.2 Hz, 1H), 5.83 (dd, *J* = 5.6, 2.8 Hz, 1H), 4.47 (dd, *J* = 5.2, 3.4 Hz, 1H), 3.55 (s, 1H), 3.42 (dd, *J* = 5.2, 1.7 Hz, 1H), 3.05 (d, *J* = 1.4 Hz, 1H), 2.01 (d, *J* = 8.5 Hz, 1H), 1.62 (dd, *J* = 8.5, 1.7 Hz, 1H); *exo*: (specific protons): *δ* 8.63 (m, 1H), 8.07 (dt, *J* = 7.8, 0.9 Hz, 1H), 7.42 (m, 1H), 7.29–7.24 (m, 4H), 6.52 (dd, *J* = 5.6, 3.1 Hz, 1H), 6.06 (dd, *J* = 5.6, 2.8 Hz, 1H), 4.15 (dd, *J* = 5.3, 1.2 Hz, 1H), 3.96 (dd, *J* = 5.2, 3.5 Hz, 1H), 3.16 (s, 1H), 3.11 (d, *J* = 1.4 Hz, 1H), 1.84 (d, *J* = 4.1 Hz, 1H), 1.48 (dd, *J* = 8.6, 1.6 Hz, 1H). ^13^C NMR (126 MHz, CDCl_3_) *endo*: *δ* 200.9, 153.4, 148.89, 143.2, 139.2, 136.9, 133.0, 131.5, 129.0, 128.4, 127.0, 122.2, 54.4, 49.2, 48.73, 48.2, 45.0; *exo*: *δ* 202.0, 153.3, 149.0, 142.2, 137.3, 136.8, 135.8, 131.6, 129.5, 127.9, 127.0, 122.4, 52.1, 49.12, 49.0, 47.0, 46.4. IR (NaCl): 2972, 1689, 1582, 1569, 1548, 1491, 1014, 670 cm^−1^.

#### 2-(3-Phenylbicyclo[2.2.1]hept-5-ene-2-carbonyl)pyridine 1-oxide (3h)^6*a*^

The product was obtained as a colorless oil (72 mg, 0.247 mmol, 99%). ^1^H NMR (400 MHz, CDCl_3_) *endo*: *δ* 8.16 (m, 1H), 7.42 (m, 1H), 7.35–7.27 (m, 6H), 7.16 (m, 1H), 6.46 (dd, *J* = 5.6, 3.2 Hz, 1H), 5.87 (dd, *J* = 5.6, 2.7 Hz, 1H), 4.50 (dd, *J* = 5.1, 3.4 Hz, 1H), 3.38 (s, 1H), 3.35 (d, *J* = 4.0 Hz, 1H), 3.09 (s, 1H), 1.88 (d, *J* = 8.6 Hz, 1H), 1.56 (ddd, *J* = 8.6, 3.5, 1.7 Hz, 1H); *exo* (specific proton): *δ* 6.41 (dd, *J* = 5.6, 3.1 Hz, 1H). ^13^C NMR (126 MHz, CDCl_3_) *endo*: *δ* 198.6, 147.4, 143.9, 140.3, 139.9, 133.1, 128.4, 127.6, 127.5, 126.3, 125.7, 125.4, 58.2, 49.1, 47.6, 46.4, 46.4; *exo*: *δ* 199.7, 147.2, 143.0, 140.4, 137.0, 136.2, 128.2, 128.0, 127.9, 126.5, 126.0, 125.6, 56.7, 48.89, 48.4, 47.9, 46.9. IR (NaCl): 2974, 1694, 1600, 1548, 1500, 1427, 1293, 1021, 852, 700, 658 cm^−1^.

#### 1-(3-Phenylbicyclo[2.2.1]hept-5-en-2-yl)ethan-1-one (3i)^40^

The product was obtained as a colorless oil (93 mg, 0.438 mmol, 88%). *Endo* product was nearly obtained as the single isomer. ^1^H NMR (400 MHz, CDCl_3_) *δ* 7.33–7.24 (m, 4H), 7.19 (m, 1H), 6.40 (dd, *J* = 5.7, 3.3 Hz, 1H), 6.03 (dd, *J* = 5.6, 2.8 Hz, 1H), 3.33 (br, 1H), 3.19 (dd, *J* = 5.0, 1.6 Hz, 1H), 3.07 (dd, *J* = 5.0, 3.3 Hz, 1H), 3.02 (d, *J* = 1.6 Hz, 1H), 2.16 (s, 3H), 1.86 (d, *J* = 8.6 Hz, 1H), 1.61 (dq, *J* = 8.6, 1.8 Hz, 1H). ^13^C NMR (100 MHz, CDCl_3_) *δ* 208.20, 144.4, 139.4, 133.1, 128.5, 128.12, 127.5, 126.0, 61.1, 48.5, 47.6, 46.5, 45.3, 29.2.

#### Bicyclo[2.2.1]hept-5-ene-2-carbonyl chloride (3j)^41^

The product was obtained as a colorless oil (47 mg, 0.300 mmol, 88%). ^1^H NMR (400 MHz, CDCl_3_) *endo*: *δ* 6.20 (dd, *J* = 5.6, 3.0 Hz, 1H), 5.99 (dd, *J* = 5.6, 2.8 Hz, 1H), 3.23 (s, 1H), 2.99 (dt, *J* = 9.4, 3.8 Hz, 1H), 2.91 (s, 1H), 1.91 (ddd, *J* = 13.0, 9.4, 3.8 Hz, 1H), 1.42 (m, 3H), 1.28 (d, *J* = 8.3 Hz, 1H); *exo* (specific protons): 6.15 (dd, *J* = 5.5, 3.0 Hz, 1H), 6.11 (dd, *J* = 5.5, 2.8 Hz, 1H). ^13^C NMR (100 MHz, CDCl_3_) *endo*: *δ* 181.2, 137.9, 132.4, 49.7, 45.7, 43.3, 42.5, 29.1.

#### 5-(Methoxycarbonyl)bicyclo[2.2.1]hept-2-ene (3k)^42^

The product was obtained as a colorless oil (677 mg, 0.891 mmol, 90%). ^1^H NMR (400 MHz, CDCl_3_) *endo*: *δ* 6.19 (dd, *J* = 5.6, 3.1 Hz, 1H), 5.93 (dd, *J* = 5.6, 2.8 Hz, 1H), 3.62 (s, 3H), 3.19 (br, 1H), 2.95 (dt, *J* = 9.3, 3.9 Hz, 1H), 2.90 (br, 1H), 1.91 (ddd, *J* = 12.7, 9.4, 3.7 Hz, 1H), 1.46–1.39 (m, 2H), 1.27 (m, 1H); *exo* (specific protons): *δ* 6.14 (dd, *J* = 5.6, 3.0 Hz, 1H), 6.10 (dd, *J* = 5.6, 3.1 Hz, 1H), 3.69 (s, 3H), 3.04 (s, 1H), 2.90 (s, 1H), 2.23 (dd, *J* = 9.7, 5.0 Hz, 1H). ^13^C NMR (126 MHz, CDCl_3_) *endo*: *δ* 175.2, 137.7, 132.3, 51.5, 49.6, 45.6, 43.1, 42.5, 29.2; *exo*: *δ* 176.7, 138.0, 135.7, 51.7, 46.5, 46.3, 42.9, 41.6, 30.3.

#### 1-(Bicyclo[2.2.1]hept-5-ene-2-carbonyl)pyrrolidin-2-one (3l)^43^

The product was obtained as a colorless oil (50 mg, 0.244 mmol, 95%). ^1^H NMR (400 MHz, CDCl_3_) *endo*: *δ* 6.19 (dd, *J* = 5.5, 3.0 Hz, 1H), 5.81 (dd, *J* = 5.5, 2.8 Hz, 1H), 3.95 (m, 1H), 3.75–3.63 (m, 2H), 3.22 (br s, 1H), 2.88 (br s, 1H), 2.58 (t, *J* = 8.1 Hz, 2H, *endo*), 2.02–1.94 (m, 2H), 1.88 (m, 1H), 1.48–1.36 (m, 3H); *exo* (specific protons): *δ* 6.13 (dd, *J* = 6.5, 3.2 Hz, 1H), 3.81–3.76 (m, 2H), 2.93 (s, 1H), 1.35–1.26 (m, 3H). ^13^C NMR (100 MHz, CDCl_3_) *endo*: *δ* 175.5, 175.0, 137.83, 131.8, 50.1, 46.1, 45.9, 44.6, 42.8, 34.0, 29.3, 17.2; *exo*: 176.9, 175.0, 138.1, 136.1, 50.1, 46.6, 45.9, 44.4, 41.9, 3.0, 29.7, 17.2. IR (NaCl): 2970, 1723, 1689, 1460, 1387, 1225, 1044, 838, 695 cm^−1^.

#### 3-(Bicyclo[2.2.2]oct-5-ene-2-carbonyl)oxazolidin-2-one (4a)^44^

The product was obtained as a colorless solid (15.1 mg, 0.068 mmol, 20%). Mp: 62–65 °C. ^1^H NMR (500 MHz, CDCl_3_) *endo*: *δ* 6.34 (m, 1H), 6.16 (t, *J* = 7.3 Hz, 1H), 4.45–4.30 (m, 2H), 3.98 (t, *J* = 8.0 Hz, 2H), 3.79–3.71 (m, 1H), 2.84 (m, 1H), 2.62 (m, 1H), 1.84 (m, 1H), 1.71 (m, 1H), 1.64 (m, 1H), 1.53 (m, 1H), 1.30–1.24 (m, 2H); *exo* (specific protons): *δ* 4.05 (m, 1H), 3.58 (m, 1H), 2.77 (m, 1H), 2.02 (m, 1H), 1.34–1.31 (m, 2H). ^13^C NMR (100 MHz, CDCl_3_) *endo*: *δ* 175.6, 153.2, 135.0, 131.3, 61.91, 42.9, 42.00, 32.8, 30.2, 29.5, 25.7, 23.9; *exo*: *δ* 135.0, 134.0, 61.82, 43.1, 41.8, 32.3, 29.7, 29.4, 27.9, 25.00. IR (NaCl): 2941, 1777, 1699, 1386, 1220, 1041, 761, 695 cm^−1^.

## Conflicts of interest

There are no conflicts to declare.

## Supplementary Material

RA-009-C9RA04098F-s001

## References

[cit1] Furuta K., Shimizu S., Miwa Y., Yamamoto H. (1989). J. Org. Chem..

[cit2] Desimoni G., Faita G., Mella M., Piccinini F., Toscanini M. (2007). Eur. J. Org. Chem..

[cit3] Evans D. A., Chapman K. T., Bisaha J. (1988). J. Am. Chem. Soc..

[cit4] Corey E. J., Imai N., Zhang H. Y. (1991). J. Am. Chem. Soc..

[cit5] Ichiyanagi T., Shimizu M., Fujisawa T. (1997). J. Org. Chem..

[cit6] Barroso S., Blay G., Pedro J. R. (2007). Org. Lett..

[cit7] Sarma D., Kumar A. (2008). Appl. Catal., A.

[cit8] Bull S. D., Davidson M. G., Johnson A. L., Mahon M. F., Robinson D. E. J. E. (2010). Chem.–Asian J..

[cit9] Suga H., Kakehi A., Mitsuda M. (2004). Bull. Chem. Soc. Jpn..

[cit10] Hiroi K., Watanabe K. (2002). Tetrahedron: Asymmetry.

[cit11] Sibi M. P., Manyem S., Palencia H. (2006). J. Am. Chem. Soc..

[cit12] Aikawa K., Irie R., Katsuki T. (2001). Tetrahedron.

[cit13] Chollet G., Rodriguez F., Schulz E. (2006). Org. Lett..

[cit14] Doherty S., Goodrich P., Hardacre C., Knight J. G., Nguyen M. T., Pârvulescu V. I., Paun C. (2007). Adv. Synth. Catal..

[cit15] Meracz I., Oh T. (2003). Tetrahedron Lett..

[cit16] Baudequin C., Baudoux J., Levillain J., Cahard D., Gaumont A.-C., Plaquevent J.-C. (2003). Tetrahedron: Asymmetry.

[cit17] Chiappe C., Malvaldi M. (2010). Phys. Chem. Chem. Phys..

[cit18] Bica K., Gaertner P. (2006). Org. Lett..

[cit19] Pinto R. M. A., Salvador J. A. R., Le Roux C. (2008). Catal. Commun..

[cit20] (b) Meindersma G. W. , Onink S. A. F. and de Haan B. A., Green Separation Processes with Ionic Liquids, in Handbook of Green Chemistry, ed. P. T. Anastas, Wiley-VCH, Weinheim, 2010, vol. 6, pp. 137–190

[cit21] Capello C., Fischer U., Hungerbühler K. (2007). Green Chem..

[cit22] Aricò F., Tundo P. (2010). Russ. Chem. Rev..

[cit23] Schäffner B., Schäffner F., Verevkin S. P., Börner A. (2010). Chem. Rev..

[cit24] Ollevier T., Desyroy V., Debailleul B., Vaur S. (2005). Eur. J. Org. Chem..

[cit25] Fürstner A. (2016). ACS Cent. Sci..

[cit26] Mayer A. C., Salit A.-F., Bolm C. (2008). Chem. Commun..

[cit27] Kanemasa S., Oderaotoshi Y., Yamamoto H., Tanaka J., Wada E., Curran D. P. (1997). J. Org. Chem..

[cit28] Howard J. A. K., Ilyashenko G., Sparkes H. A., Whiting A. (2007). Dalton Trans..

[cit29] (b) Spiller G. A. , in Basic Metabolism and Physiological Effects of the Methylxanthines in Caffeine, ed. G. A. Spiller, CRC press LLC, Boca Raton, Florida, 1st edn, 1997, ch. 10, pp. 199–255

[cit30] Luo F.-T., Lo H.-K. (2011). J. Organomet. Chem..

[cit31] Reichardt C. (1994). Chem. Rev..

[cit32] The loss of catalyst during its recovery process could have been the cause.

[cit33] Other previously listed solvents, *e.g.* NMP, EtOAc, and acetone, did not lead to improved yields.

[cit34] Erfurt K., Wandzik I., Walczak K., Matuszek K., Chrobok A. (2014). Green Chem..

[cit35] Blakesley D. W., Payne S. C., Hagen K. S. (2000). Inorg. Chem..

[cit36] Grommet A. B., Bolliger J. L., Browne C., Nitschke J. R. (2015). Angew. Chem., Int. Ed..

[cit37] Matson E. M., Bertke J. A., Fout A. R. (2014). Inorg. Chem..

[cit38] Kascatan-Nebioglu A., Panzner M. J., Garrison J. C., Tessier C. A., Youngs W. J. (2004). Organometallics.

[cit39] Li Y., Wang C., Hao J., Cheng M., Jia G., Li C. (2015). Chem. Commun..

[cit40] Singh R. S., Adachi S., Tanaka F., Yamauchi T., Inui C., Harada T. (2008). J. Org. Chem..

[cit41] Mukhina O. A., Bhuvan Kumar N. N., Arisco T. M., Valiulin R. A., Metzel G. A., Kutateladze A. G. (2011). Angew. Chem., Int. Ed..

[cit42] Matuszek K., Coffie S., Chrobok A., Swadźba-Kwaśny M. (2017). Catal. Sci. Technol..

[cit43] Sibi M. P., Chen J., Stanley L. (2007). Synlett.

[cit44] Evans D. A., Barnes D. M., Johnson J. S., Lectka T., von Matt P., Miller S. J., Murry J. A., Norcross R. D., Shaughnessy E. A., Campos K. R. (1999). J. Am. Chem. Soc..

