# Supplementary material for: Recyclable iron(ii) caffeine-derived ionic salt catalyst in the Diels–Alder reaction of cyclopentadiene and α,β-unsaturated N-acyl-oxazolidinones in dimethyl carbonate
Source: RSC Adv. 2019 Jul 15;9(38):21956–63. doi: 10.1039/c9ra04098f (PMC9066430; doi:10.1039/c9ra04098f)
Supplement: RA-009-C9RA04098F-s001 [file RA-009-C9RA04098F-s001.pdf]

Electronic Supplementary Material (ESI) for RSC Advances.
This journal is © The Royal Society of Chemistry 2019

Supplementary Information for

Recyclable Iron(II) Caffeine-derived Ionic Salt Catalyst in the Diels-Alder Reaction of Cyclopentadiene and α,β-Unsaturated *N*-Acyl-oxazolidinones in Dimethyl Carbonate

Di Meng, Dazhi Li, and Thierry Ollevier*

Département de chimie, Université Laval, 1045 avenue de la Médecine, Québec, QC, G1V 0A6, Canada

E-mail: thierry.ollevier@chm.ulaval.ca

Table of content

1. Interaction between xanthinium and $Fe(OTf)_2$ 2
2. ^{1}H and ^{19}F NMR of C1 before and after the catalytic reactions 8
3. Comparison between C1–C4 9
4. Copies of ^{1}H NMR and ^{13}C NMR spectra of the products 11
5. References 23

1. Interaction between xanthinium and $Fe(OTf)_2$

As far as we are concerned, there has been no study of the interaction between xanthinium and $Fe(OTf)_2$ by spectra reported to date. For this purpose, the interaction between xanthinium and $Fe(OTf)_2$ was studied by using UV-Vis, FTIR, ^1H, ^{13}C and ^{19}F NMR, and HRMS. The UV-Vis spectra of xanthinium are shown in Figure S1. The xanthinium possesses two main absorbance bands at 204 nm (absorption coefficient 1.3×10^4, π-π^* transition) and 268 nm (absorption coefficient 7.0×10^3, n-π^* transition), and no absorbance in the range of 300–800 nm. Upon addition of different equivalents of $Fe(OTf)_2$ (0.2 to 10 equiv.) to the solution, the UV-Vis spectra of xanthinium did not show significant changes neither at 204 nm nor 268 nm, providing no clear trend of change. Since the UV-Vis spetra must be recorded at low concentration (5×10^{-5} mol/L), the interaction between xanthinium and $Fe(OTf)_2$ was further diminished, and finally not detectable by the spectra. Thus, the UV-Vis spetra was considered not suitable for the study of the interaction between xanthinium and $Fe(OTf)_2$. It is very important to note that, in a specific type of spectrum, no observation of changes does not necessarily mean no interaction between two species, because the spectrum technique might be not sensitive enough to detect the changes.

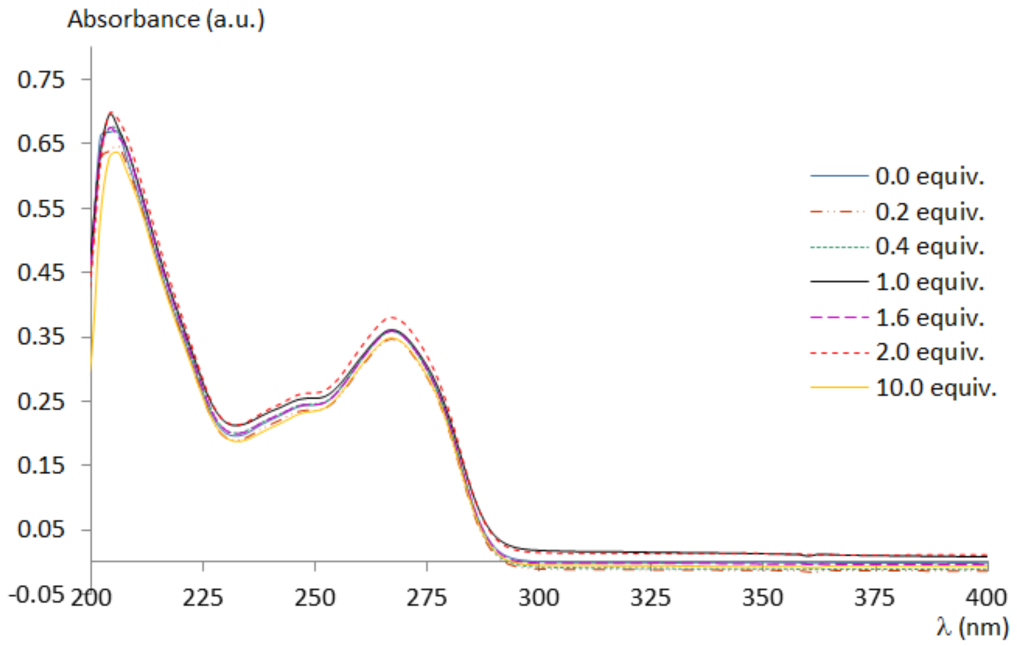

Figure S1. UV-Vis spectra of xanthinium **C1** (5×10^{-5} mol/L in EtOH) upon addition of different equivalents of $Fe(OTf)_2$) at 25 °C. The corresponding $Fe(OTf)_2$ solution without **C1** was used as reference.

In addition to UV-Vis spectra, FTIR spectra were studied. The FTIR spectrum of xanthinium of **C1** shows two carbonyl signals at 1723 cm^{-1} and 1683 cm^{-1} (symmetric and antisymmetric stretching). After mixing with $Fe(OTf)_2$, the signals shifted to 1726 cm^{-1} and 1676 cm^{-1}, respectively (Figure S2). The peaks at 1194 (C–F stretching, very strong), 622 and 608 cm^{-1} also shifted to 1209, 641 and 618 cm^{-1}, respectively. On the other hand, the signal intensity at 1140 cm^{-1} increased, while the signal at 855 cm^{-1} almost disappeared. The changes of C=O and C–F stretching frequencies (corresponding to xanthinium cation and $N(SO_2CF_3)_2^-$ anion, respectively) in FTIR spectra indicated that interactions between xanthinium and $Fe(OTf)_2$ may involve coordination of Fe(II) to carbonyl groups.

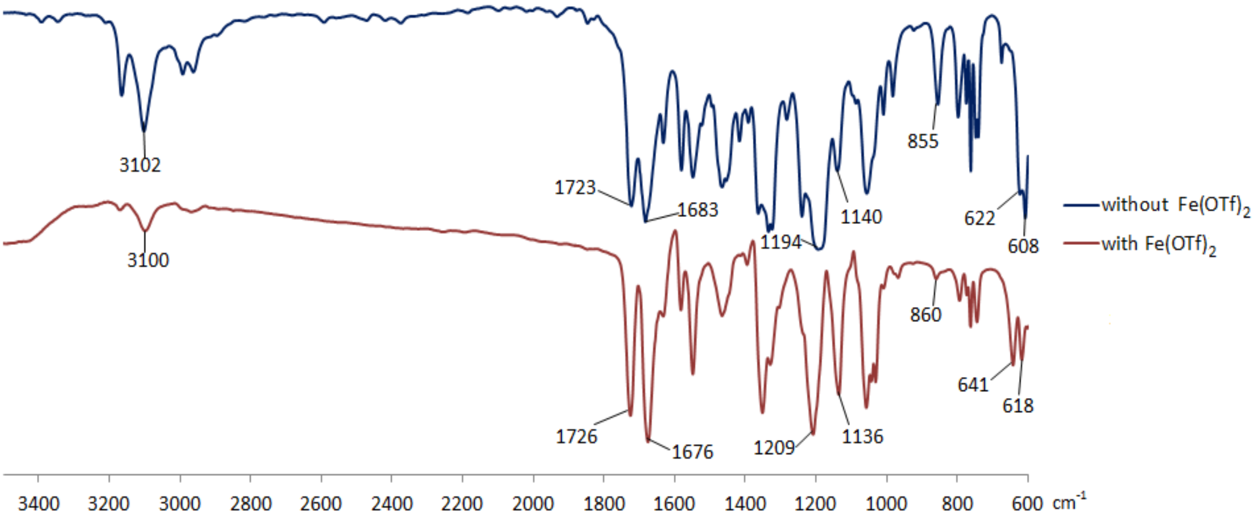

Figure S2. FTIR spectra of xanthinium of **C1** without the presence of $Fe(OTf)_2$ and in the presence of $Fe(OTf)_2$ (1.0 equiv., the signals of $Fe(OTf)_2$ have been subtracted from the spectra).

Subsequently, the NMR spectra were used to study the interaction between xanthinium and $Fe(OTf)_2$ (Figure S3, S4 and S5). As shown in Figure S3, upon addition of additional equivalents of $Fe(OTf)_2$ to xanthinium, the chemical shifts of ^1H NMR showed no obvious changes. This indicated that the interaction with $Fe(OTf)_2$ in solution did not cause any significant changes of the ^1H magnetic environment in the xanthinium. Thus, ^1H NMR was not suitable for the study of the interaction between xanthinium and $Fe(OTf)_2$. In addition, ^{13}C NMR was run (Figure S4). Upon addition of $Fe(OTf)_2$, all signals of xanthinium shifted upfield by 1.21–1.46 ppm (more electron enriched). Notably, the signal-to-noise ratio of carbon C3 and C6 increased, while the signals of C8 and C10 decreased.

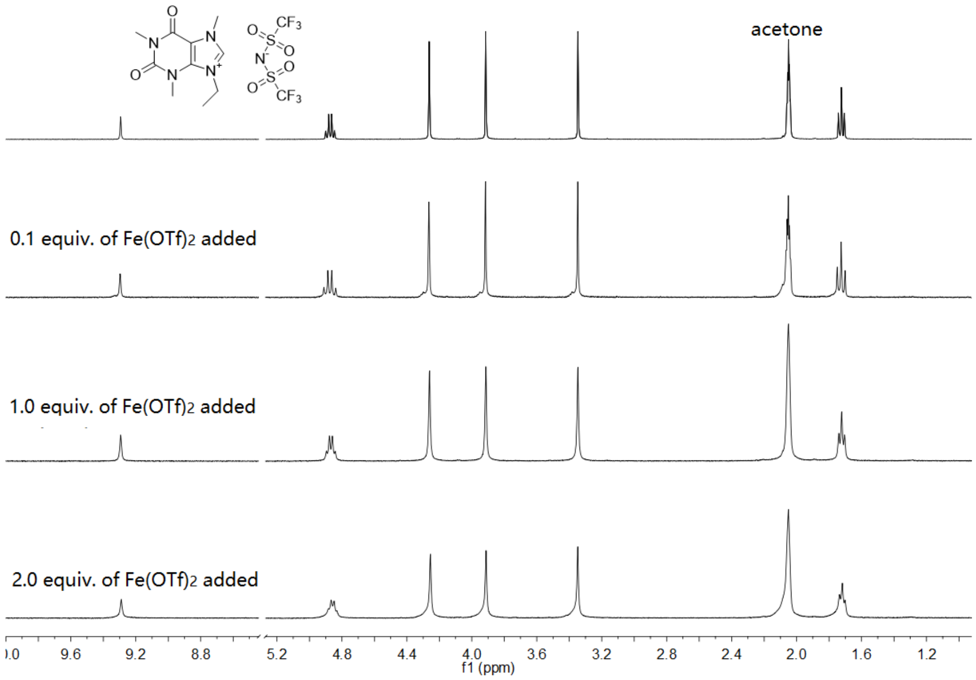

Figure S3. ^{1}H NMR spectra of xanthinium (5 mg/mL in $(CD_3)_2CO$, 400 MHz) upon addition of different equivalents of $Fe(OTf)_2$ at 25 °C.

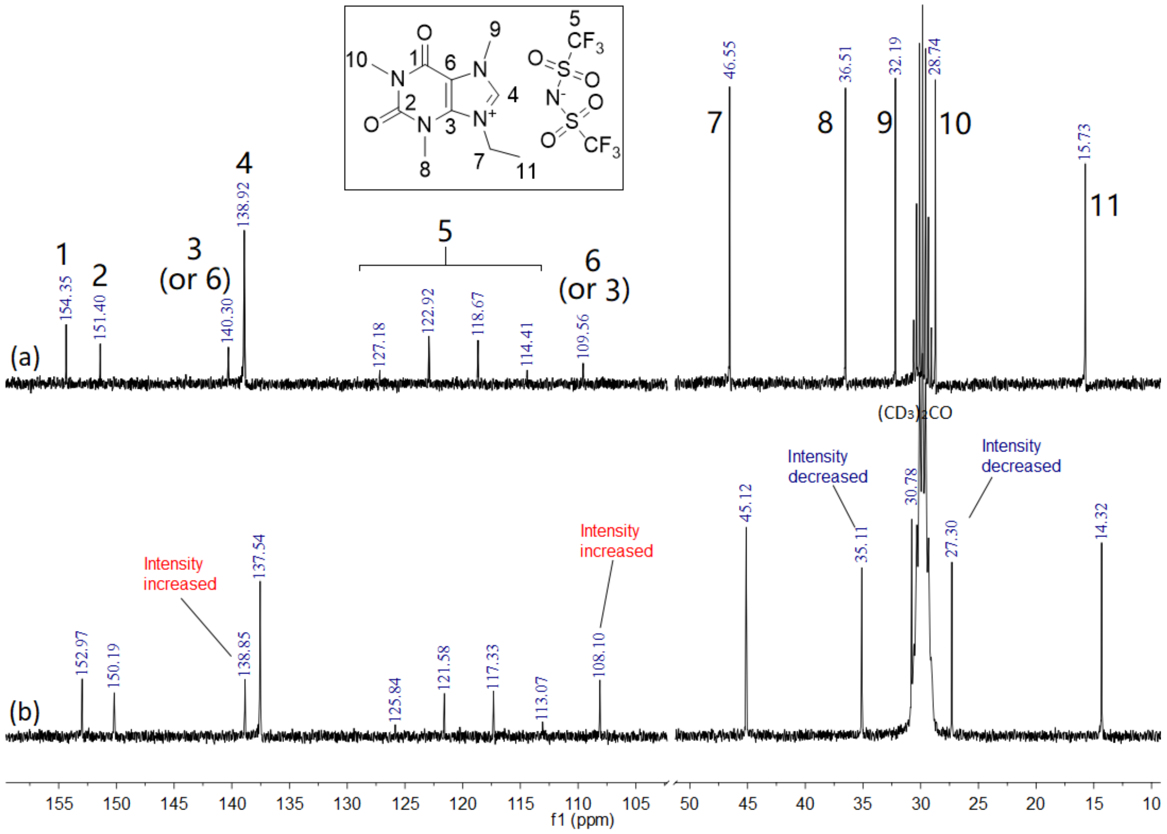

Figure S4. ^{13}C NMR spectra of (a) xanthinium (0.27 mol/L) and (b) xanthinium+$Fe(OTf)_2$ (xanthinium = 0.27 mol/mL; $Fe(OTf)_2$ = 0.027 mol/mL) in $(CD_3)_2CO$ (75 MHz) shown at 10–50 ppm and 105–155 ppm using solvent signal as reference.

Furthermore, the ^{19}F NMR spectra were recorded as well (Figure S5). The ^{19}F NMR signals for NTf_2^- of xanthinium and OTf^- of $Fe(OTf)_2$ were recorded, and clearly showed at –79.99 ppm and –56.91 ppm (broad peak) respectively. Compared to ionic salts of OTf^- (^{19}F NMR of $Ca(OTf)_2$ and $Bi(OTf)_3$ appears at –79.24 ppm and –79.00 ppm,[1] respectively), the signal of pure $Fe(OTf)_2$ shifts to much deshielded region, implying that the triflate groups in $Fe(OTf)_2$ are more electron-deficient than free ionic OTf^-. Thus, the triflate groups in $Fe(OTf)_2$ were considered coordinating to Fe(II) (donating the electrons to Fe^{2+} center) in $(CD_3)_2CO$ without the presence of other ligand. Upon addition of 0.1 equiv. of $Fe(OTf)_2$ to xanthinium, the signal of triflate shifted to a much shielded value of –73.40 ppm, closer to the ionic OTf^-. This chemical shift was also comparable with the value of unbound OTf^- (–78.96 ppm,[2] –79.59 ppm[3] and –69.7 ppm[4]), which was liberated from $Fe(OTf)_2$ after the Fe^{2+} coordinating to a ligand. For this reason, most of Fe^{2+} was considered coordinating to xanthinium when the ratio of xanthinium to $Fe(OTf)_2$ was high. After 0.5 equiv. of $Fe(OTf)_2$ was added to xanthinium, the signal of triflate was hardly detected due to the broadening of

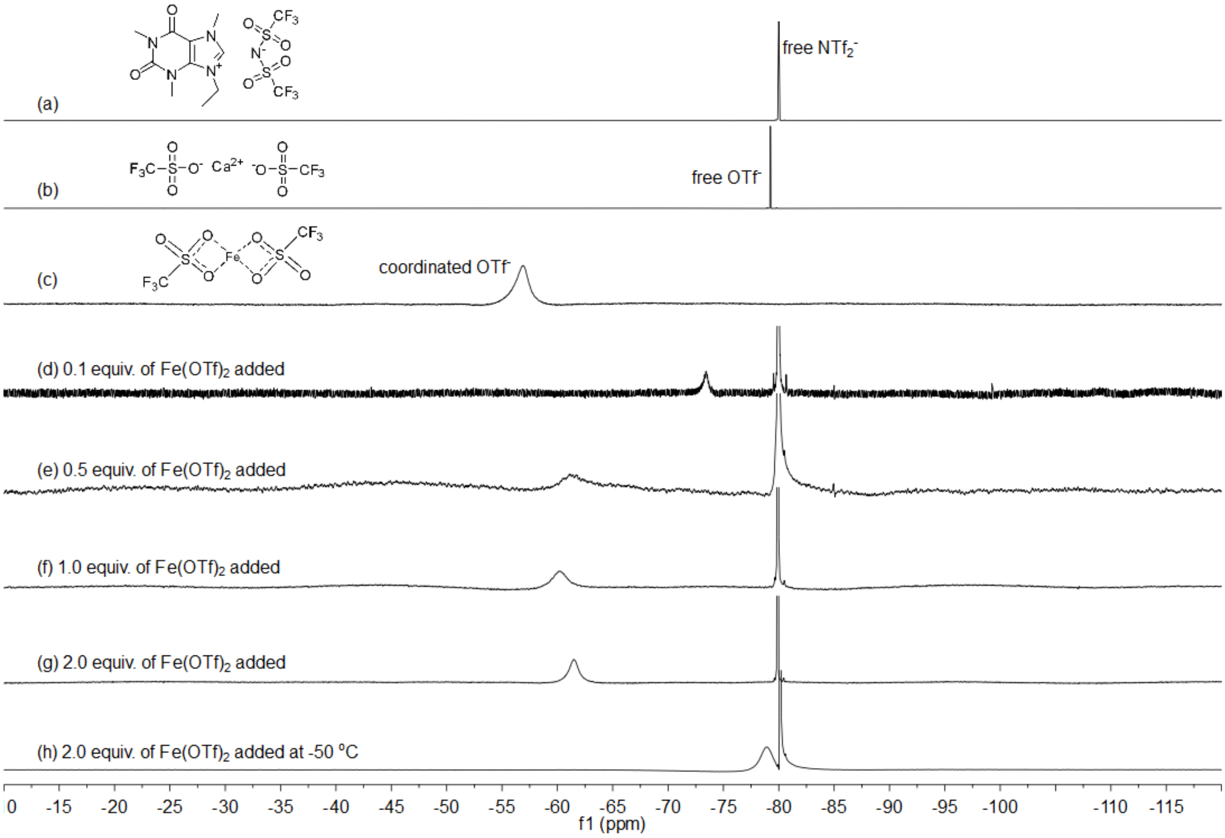

Figure S5. ^{19}F NMR spectra of (a) xanthinium, (b) $Ca(OTf)_2$, (c) $Fe(OTf)_2$, (d)-(f) xanthinium upon addition of different equivalents of $Fe(OTf)_2$ at 25 °C and (g) xanthinium upon addition of 2.0 equivalents of $Fe(OTf)_2$ at –50 °C ($(CD_3)_2CO$, 376 MHz, 5 mg/mL of xanthinium)

the spectrum. Upon addition of 1.0 equiv. and 2.0 equiv. of $Fe(OTf)_2$ to xanthinium, the signals of triflate shifted to –60.19 and –61.47 ppm (Figure S5(f) and S5(g)), respectively, showing an obvious trend closer to $Fe(OTf)_2$. This change was due to the high content of $Fe(OTf)_2$ which was not able to fully coordinate to xanthinium. On the other hand, since the NTf_2^- is not coordinating (remaining as free anion), the signals of NTf_2^- only showed slight changes from –79.99 ppm to –79.90 ppm. In order to slow down the interaction, the ^{19}F NMR was recorded at –50 °C subsequently (Figure 5(h)). The signal of triflate shifted to –78.94 ppm (close to –79 ppm of ionic OTf^-), indicating that the triflate was "frozen" as a free anion. Although the chemical shift of triflate at –50 °C was close to free OTf^-, the shape of peak was still broadened due to the slow dynamic ion exchange.

Finally, high resolution mass spectrometry (HRMS) was used to study the solution of **C1** (Figure S6 and S7). Although the Fe^{2+}-containing peak was not detected by HRMS, the

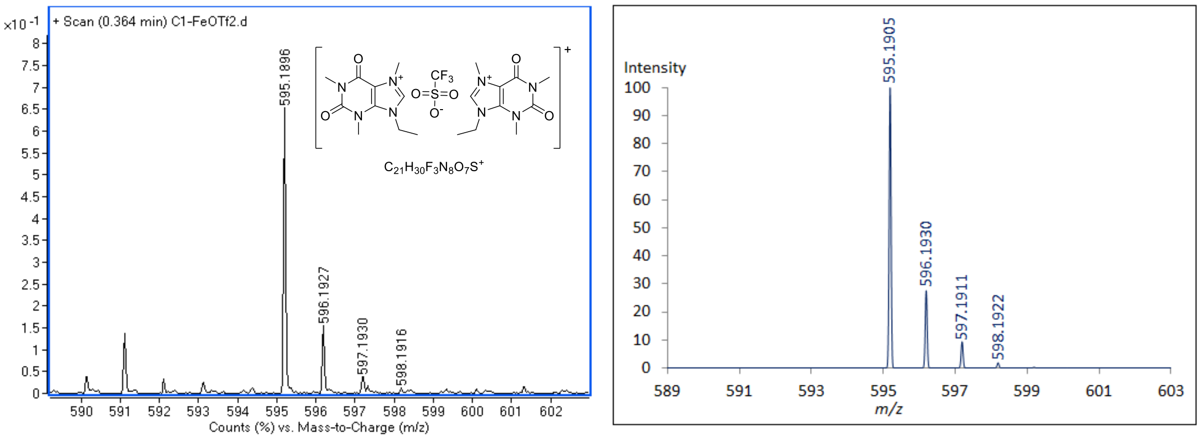

(a) experimental isotopic distribution (b) calculated isotopic distribution

Figure S6. HRMS signal of a [2xanthinium+OTf]$^+$ species (ESI-TOF positive ion mode).

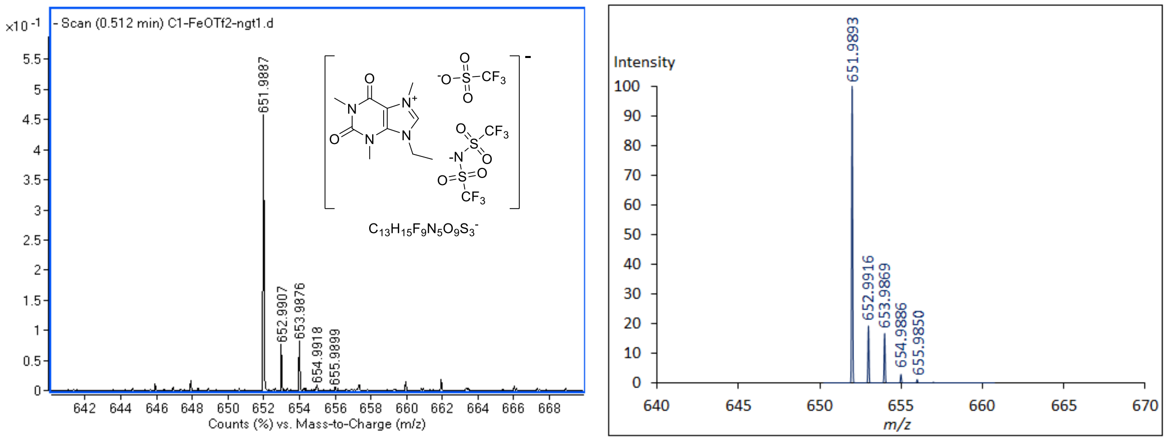

(a) experimental isotopic distribution (b) calculated isotopic distribution

Figure S7. HRMS signal of a [xanthinium+OTf+NTf$_2$]$^-$ species (ESI-TOF negative ion mode).

signals containing xanthinium species from ion exchange were detected. A $[2xanthinium+OTf]^+$ (found 595.1896, diff. = −1.5 ppm) species was found in positive ion mode (Figure S6). The exact mass and the isotopic distribution confirmed the existence of this species unambiguously. When running in negative ion mode, a $[xanthinium+OTf+NTf_2]^−$ (found 651.9887, diff. = −0.9 ppm) species was detected, confirming by the exact mass and isotopic distribution (Figure S7). In addition, other species, such as $[2xanthinium+NTf_2]^+$ and $[xanthinium+2NTf_2]^−$ were detected by HRMS as well. According to the HRMS, the ion exchange between xanthinium-NTf_2 and $Fe(OTf)_2$ is simply proposed in Scheme S1. During the process of ion exchange, free Fe^{2+} and OTf^- could be released and transferred between different species. Thus, the Lewis acidic free Fe^{2+} could bind with the carbonyl of dienophile, activating the substrates in the D-A reaction.

The detectable changes of FTIR, ^{13}C and ^{19}F NMR, and HRMS spectra clearly showed that the interactions between xanthinium and $Fe(OTf)_2$ do exist. According to FTIR, and ^{13}C and ^{19}F NMR, Fe^{2+} could possibly coordinate to the carbonyls of xanthinium; while the HRMS confirmed the ion exchange between xanthinium and $Fe(OTf)_2$.

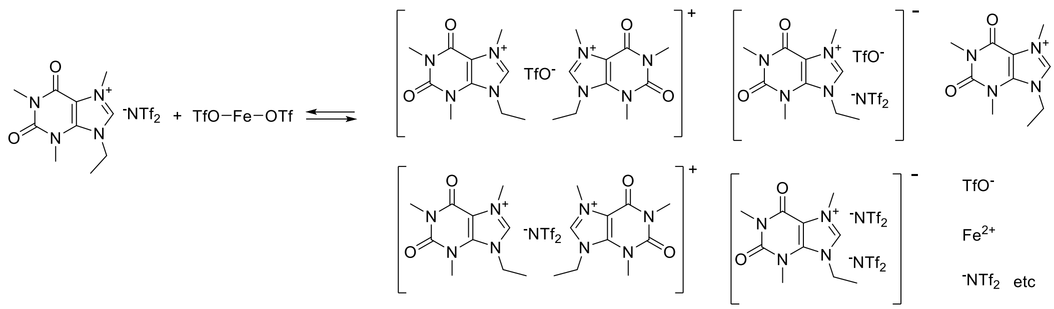

Scheme S1. Ion exchange between xanthinium-NTf_2 and $Fe(OTf)_2$.

2. ^{1}H and ^{19}F NMR of C1 before and after the catalytic reactions

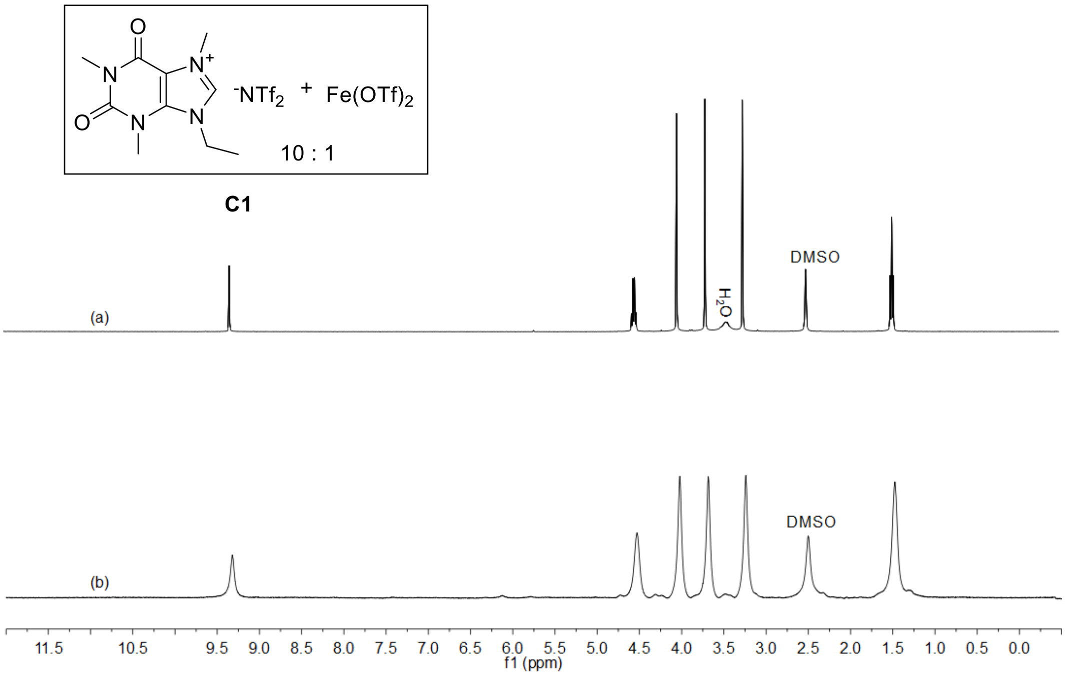

Figure S8. ^{1}H NMR of **C1**: (a) before and (b) after catalytic reactions (400 MHz, $(CD_3)_2SO$)

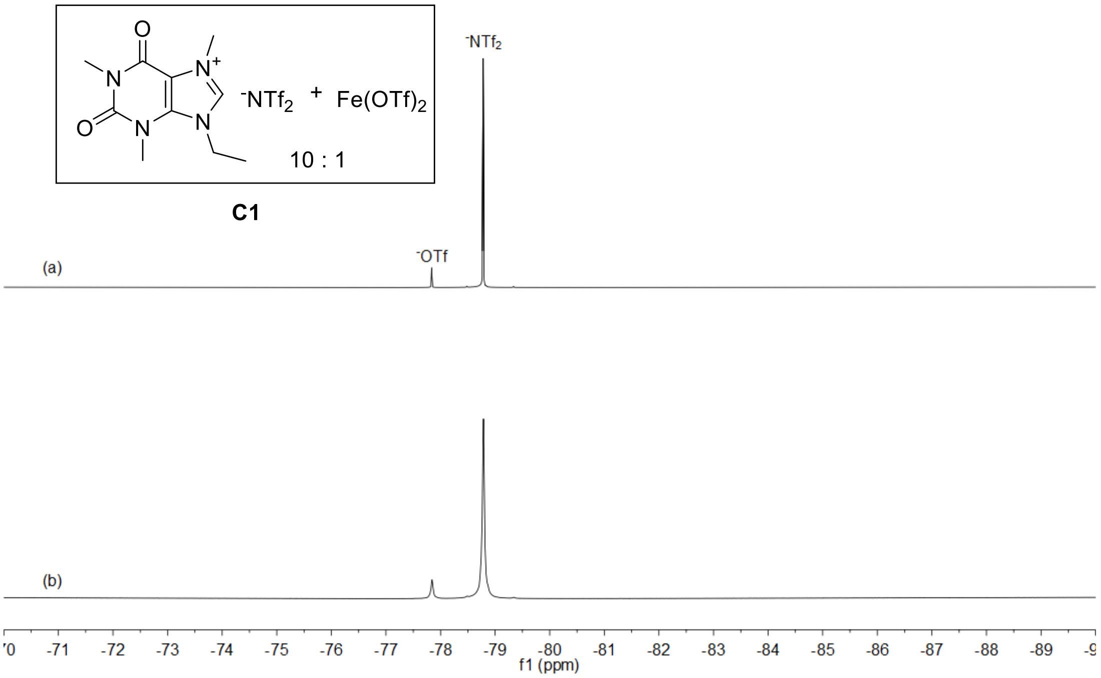

Figure S9. ^{19}F NMR of **C1**: (a) before and (b) after catalytic reactions (376 MHz, $(CD_3)_2SO$)

3. Comparison between C1–C4

The change of Gibbs free energy and Mulliken charge of Fe(II) or Fe(III) after the ion exchange were calculated at B3LYP/6-31G/LANL2DZ level in gas phase to obtain an over-simplified evaluation for **C1**–**C4**. The results are shown in Scheme S2.

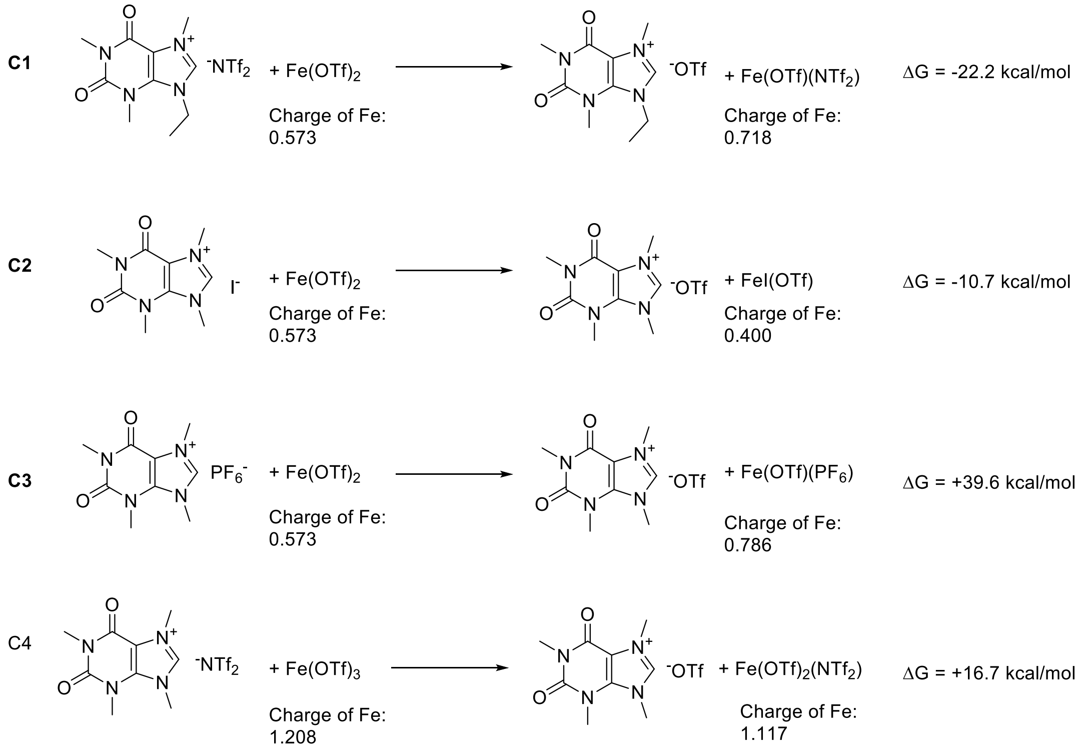

Scheme S2. Ion exchange between xanthiniums and Fe salts. Gibbs energies and Mulliken charges were calculated at B3LYP/6-31G/LANL2DZ level.

According to the calculations, the ion exchange in **C1** is favored (ΔG = –22.2 kcal/mol) which is supported by the ^{19}F NMR change and HRMS. After ion exchange, the Mulliken charge of $Fe(OTf)_2$ (0.573) increases to 0.718 of $Fe(OTf)(NTf_2)$, which indicates a more Lewis acidic Fe(II) salt. Thus, **C1** resulted in excellent yields on catalyzing the D-A reactions (main text Table 1, entry 6). In **C2**, the ion exchange is expected to be favored (ΔG = –10.7 kcal/mol), and this is consistent to the coordinating nature of $I^–$ (easiness of binding to transition metals). However, the ion exchange in **C2** is expected to result in less Lewis acidic FeI(OTf) (Mulliken charge drops from 0.573 to 0.400). Thus, **C2** afforded low yields of products (33% yield, main text Table 1, entry 7). In **C3**, the ion exchange is expected not to be favourable (ΔG = +39.6 kcal/mol), and in fact the reversed reaction was spontaneous and already known in the literature.[5] Thus, the xanthinium and $Fe(OTf)_2$ in **C3** remained essentially unchanged, and the yield using **C3** (55% yield, Table 1, entry 8) is lower than

the yield using $Fe(OTf)_2$ alone (81% yield, Table 1, entry 11) by 26%. In **C4**, the ion exchange is expected to be not favourable (ΔG = +16.7 kcal/mol) either. Since $Fe(OTf)_3$ is very Lewis acidic (1.208 of Mulliken charge), excellent yield (97% yield, Table 1, entry 9) was still obtained, which is comparable to the yield using **C1**.

4. Copies of ^1H NMR and ^{13}C NMR spectra of the products

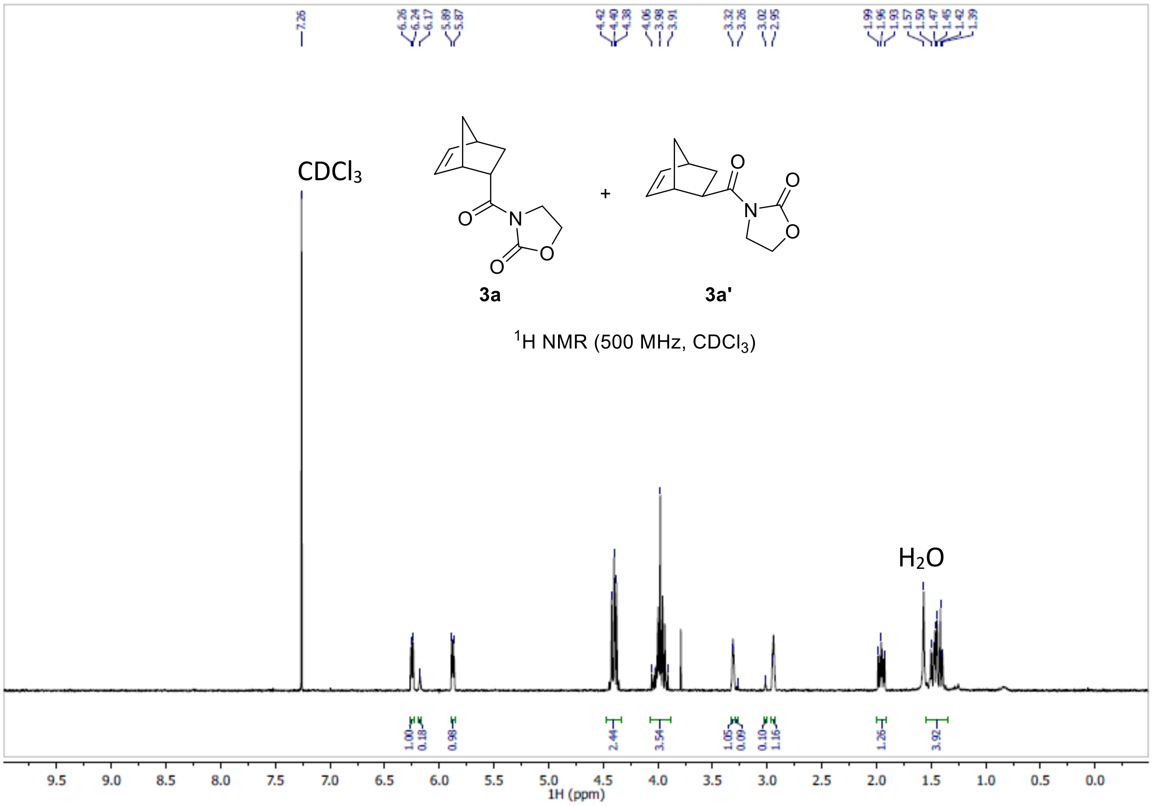

^1H NMR of **3a+3a'** (500 MHz, $CDCl_3$, mixture of *endo*/*exo* products)

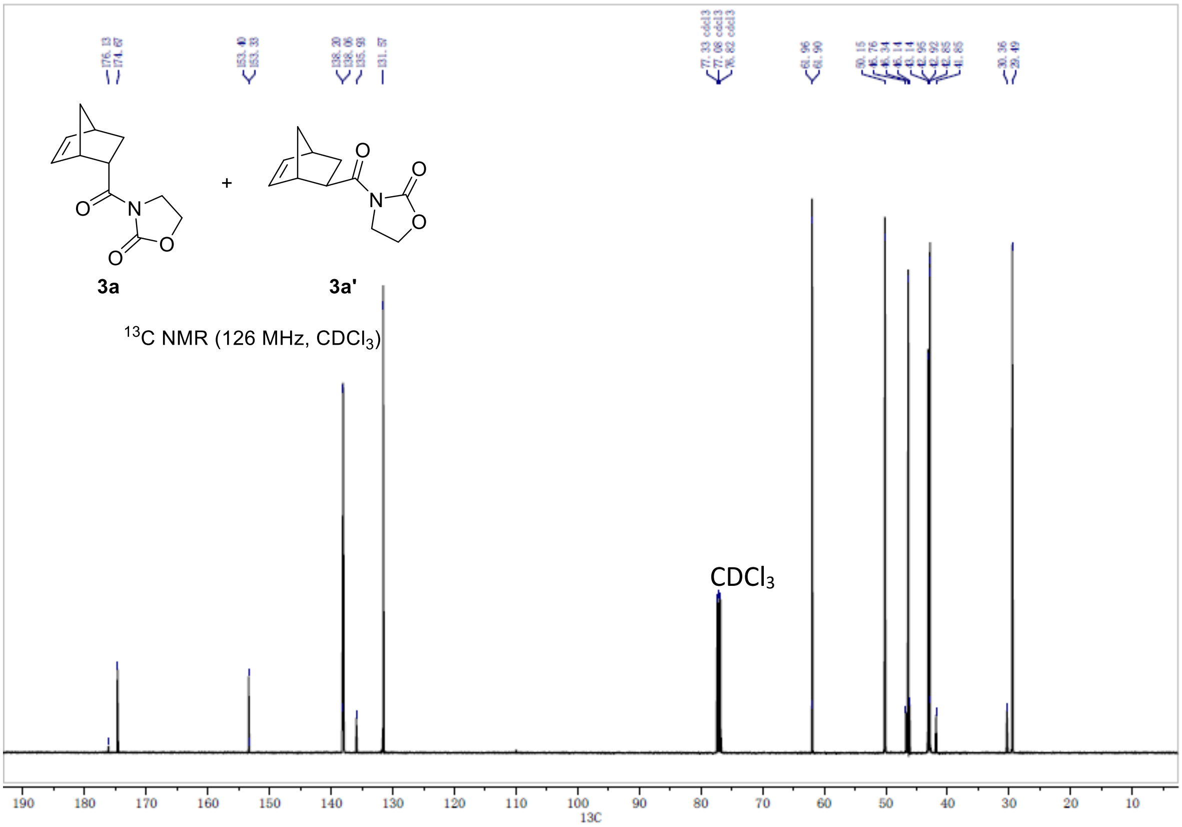

^{13}C NMR of **3a+3a'** (126 MHz, $CDCl_3$, mixture of *endo*/*exo* products)

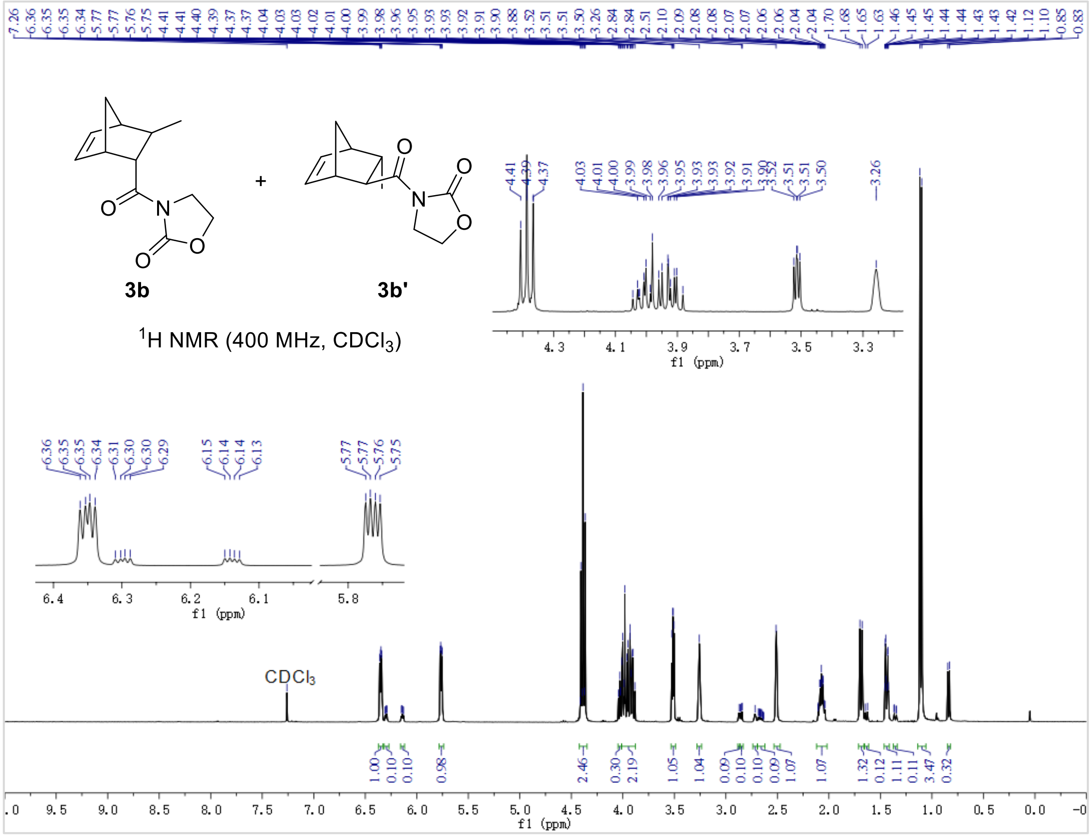

^{1}H NMR of **3b+3b'** (400 MHz, $CDCl_3$, mixture of *endo*/*exo* products)

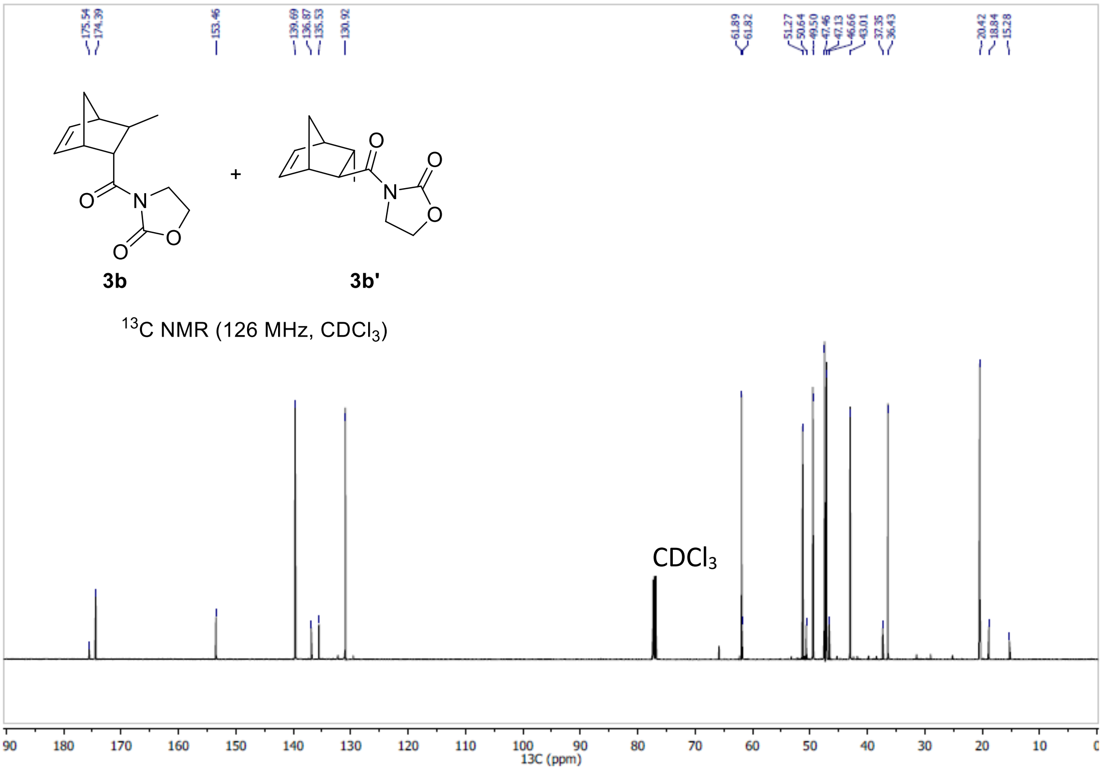

^{13}C NMR of **3b+3b'** (126 MHz, $CDCl_3$, mixture of *endo*/*exo* products)

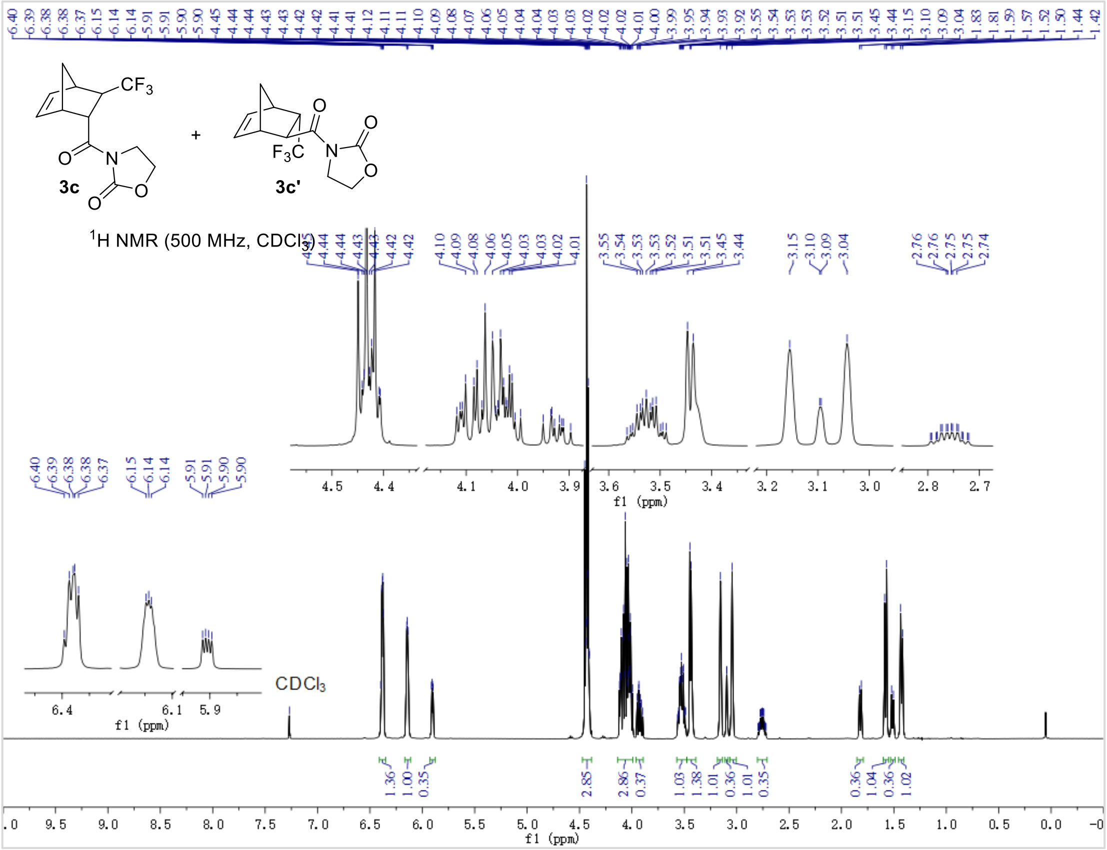

^{1}H NMR of **3c+3c'** (500 MHz, $CDCl_3$, mixture of *endo*/*exo* products)

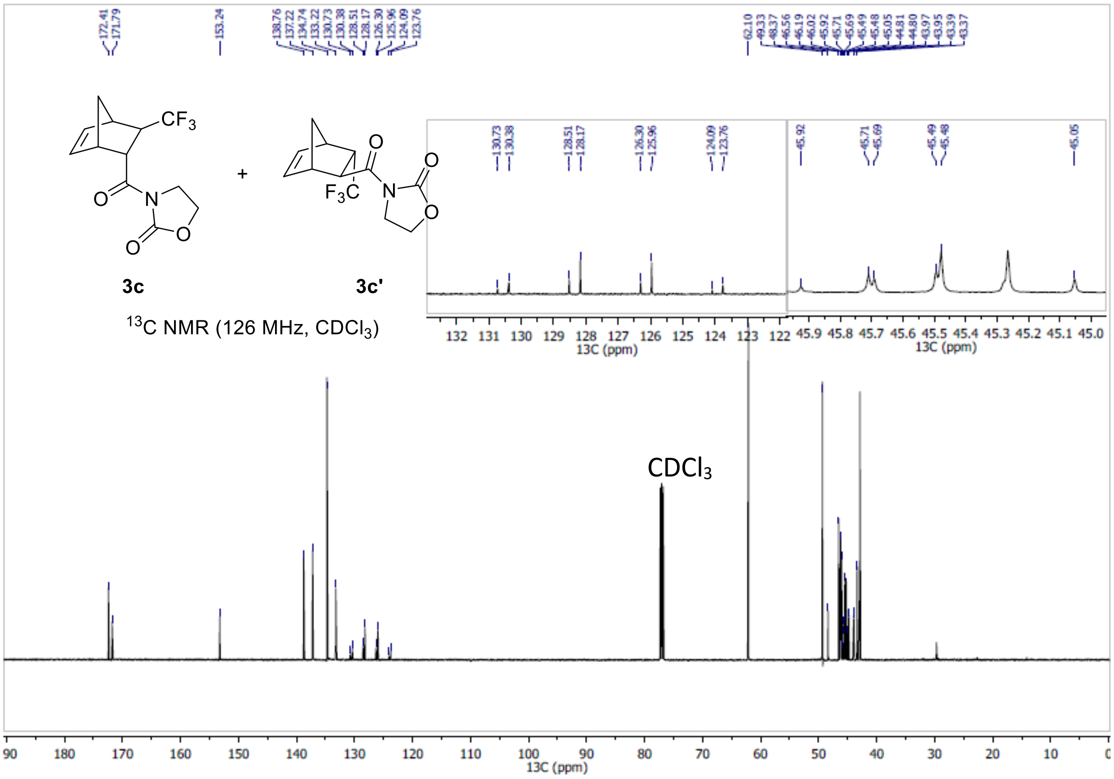

^{13}C NMR of **3c+3c'** (126 MHz, $CDCl_3$, mixture of *endo*/*exo* products)

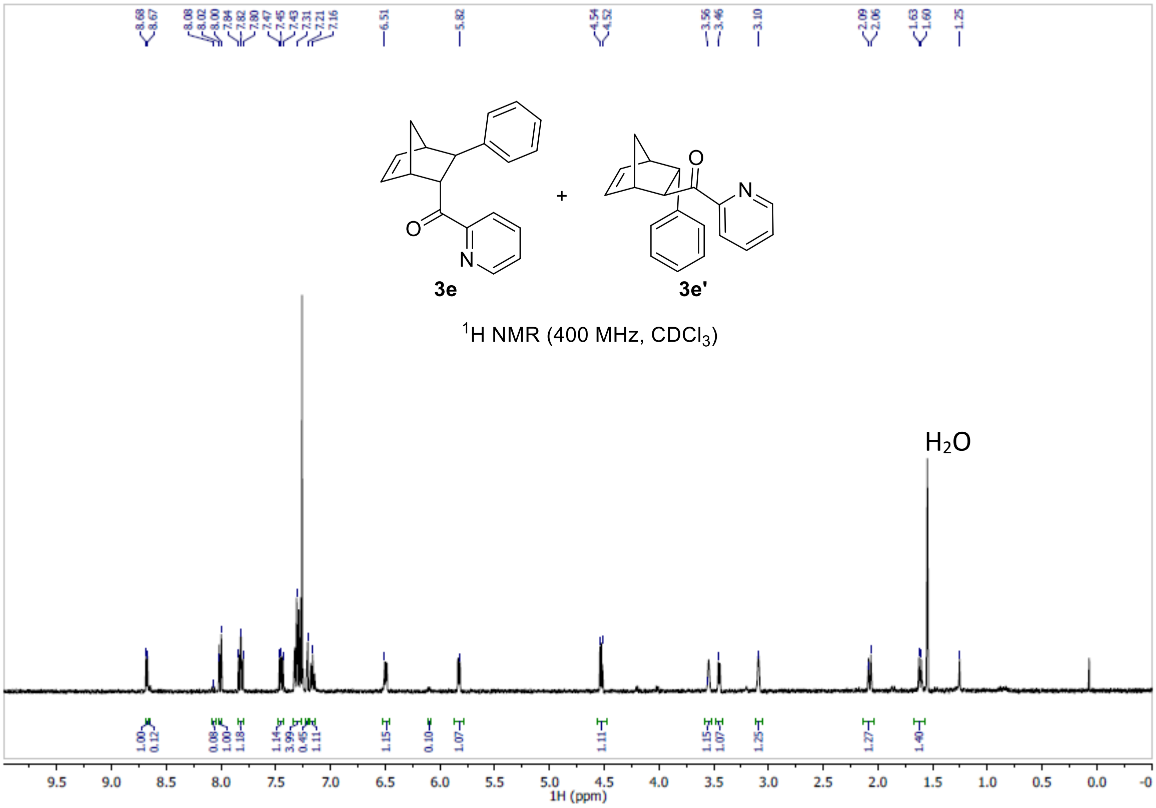

^{1}H NMR of **3e+3e'** (400 MHz, $CDCl_3$, mixture of *endo*/*exo* products)

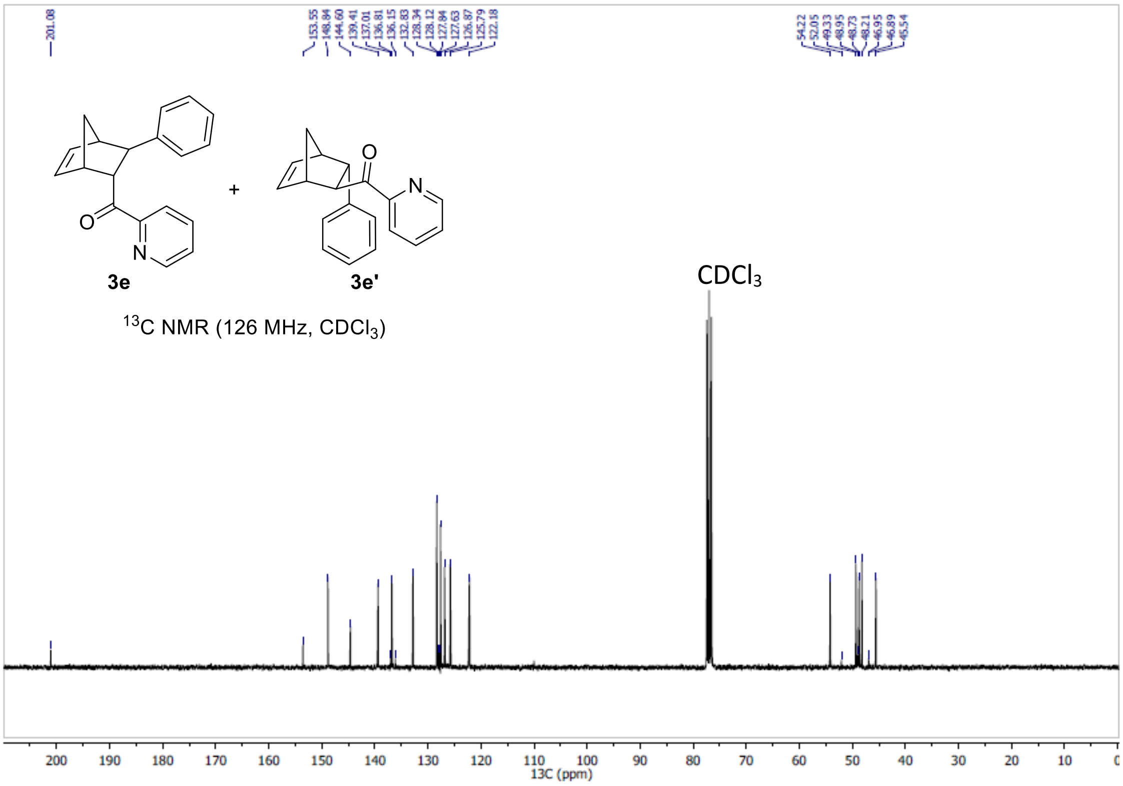

^{13}C NMR of **3e+3e'** (126 MHz, $CDCl_3$, mixture of *endo*/*exo* products)

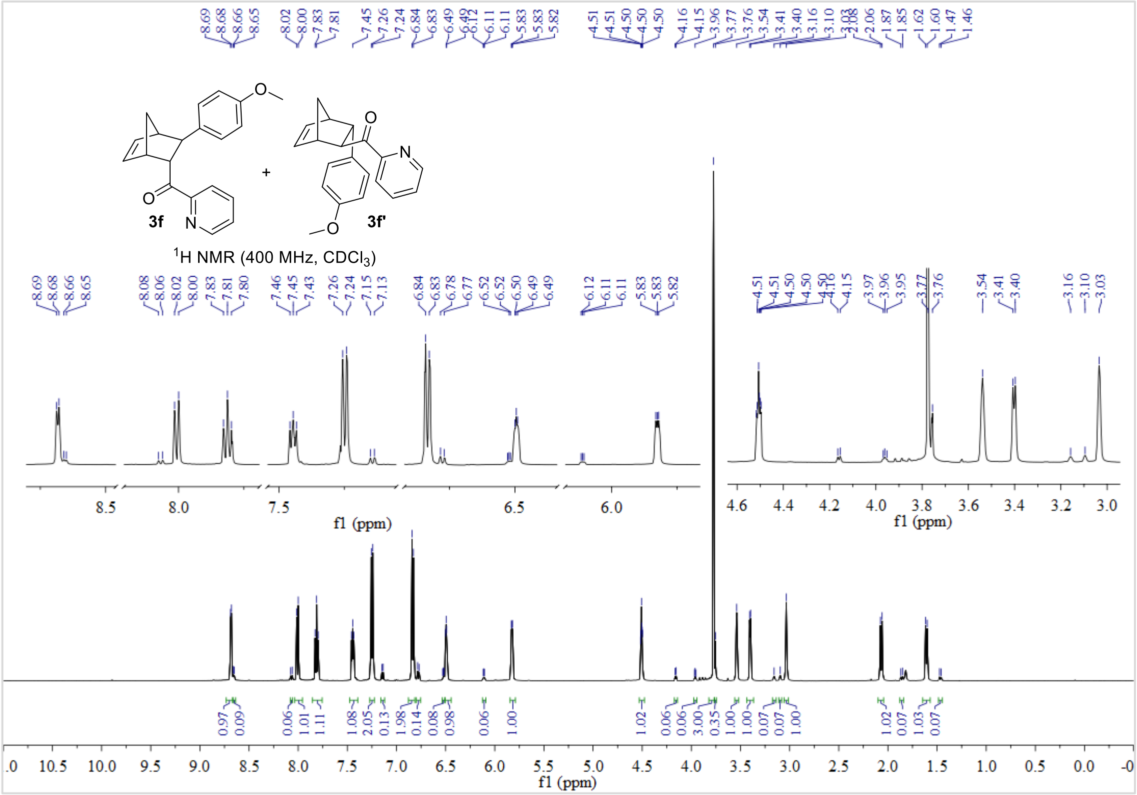

^{1}H NMR of **3f+3f’** (400 MHz, $CDCl_3$, mixture of *endo*/*exo* products)

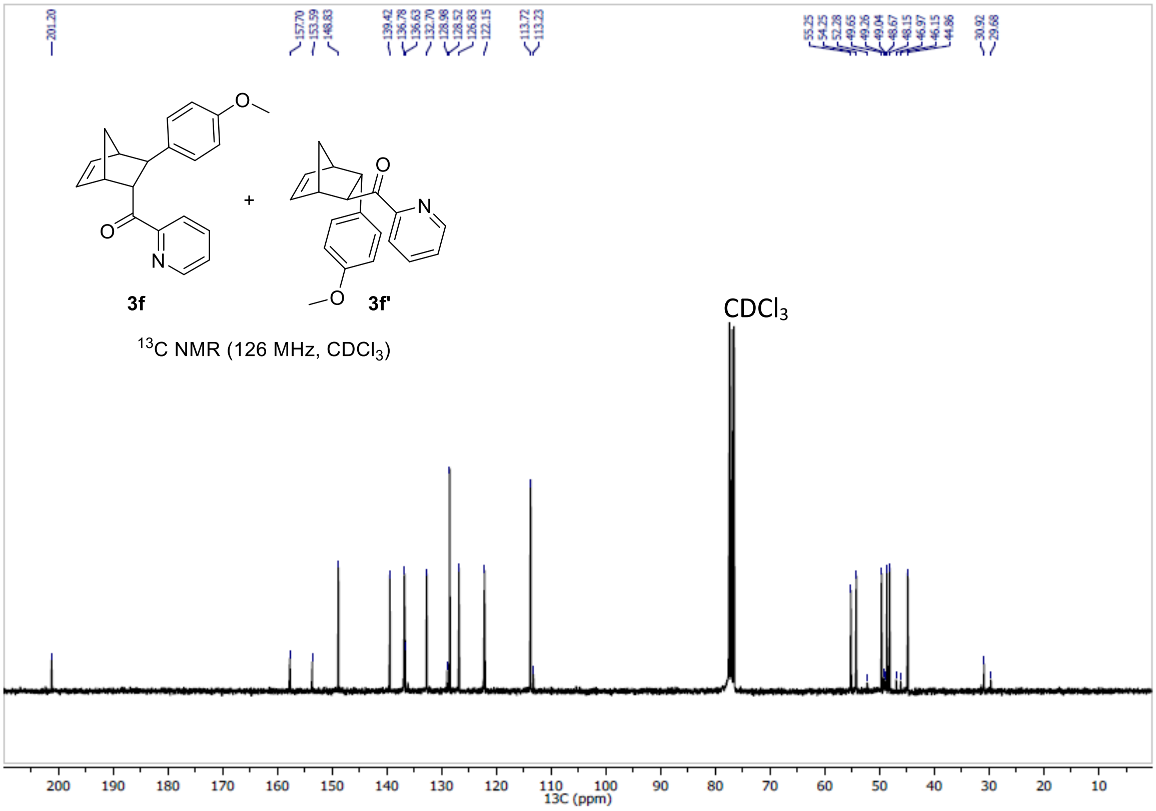

^{13}C NMR of **3f+3f’** (126 MHz, $CDCl_3$, mixture of *endo*/*exo* products)

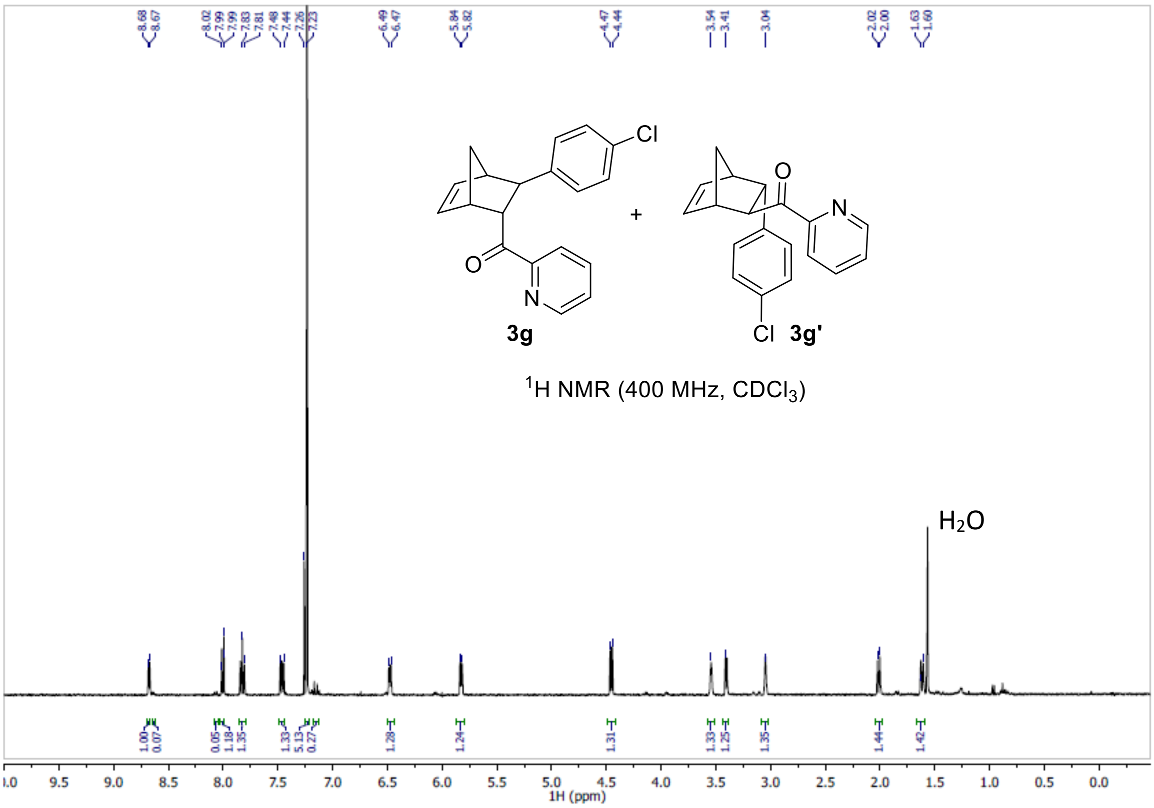

^{1}H NMR of **3g+3g’** (400 MHz, $CDCl_3$, mixture of *endo*/*exo* products)

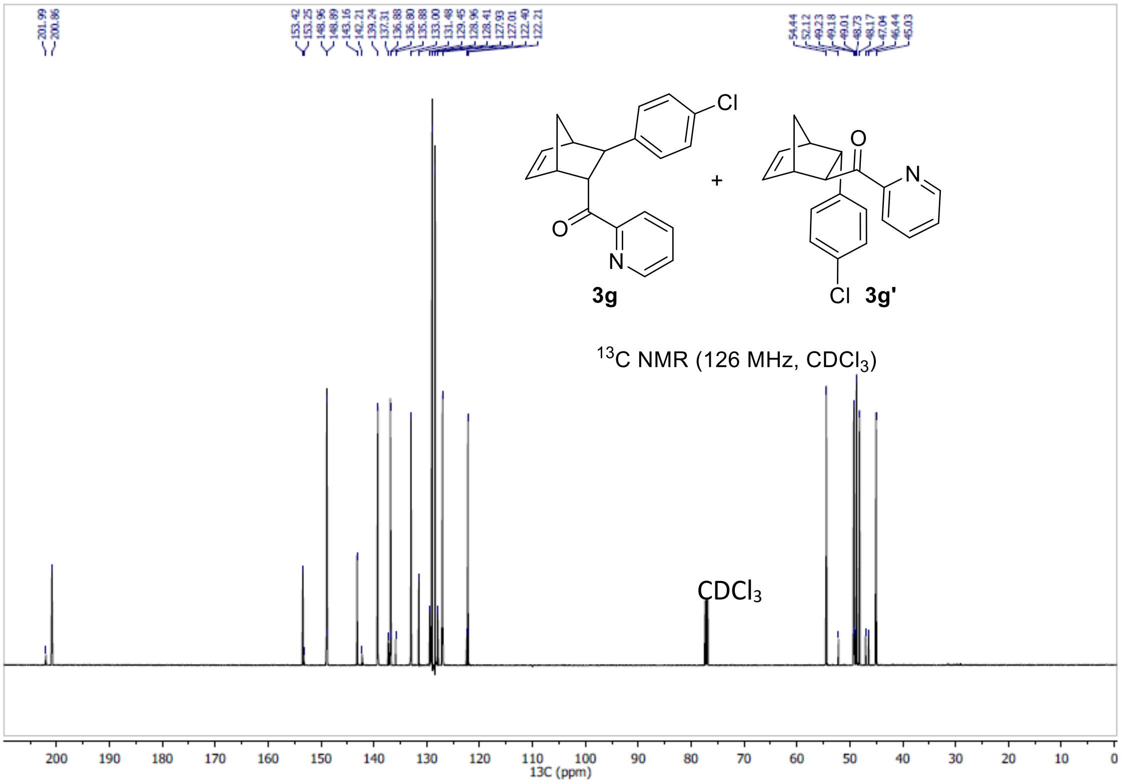

^{13}C NMR of **3g+3g’** (126 MHz, $CDCl_3$, mixture of *endo*/*exo* products)

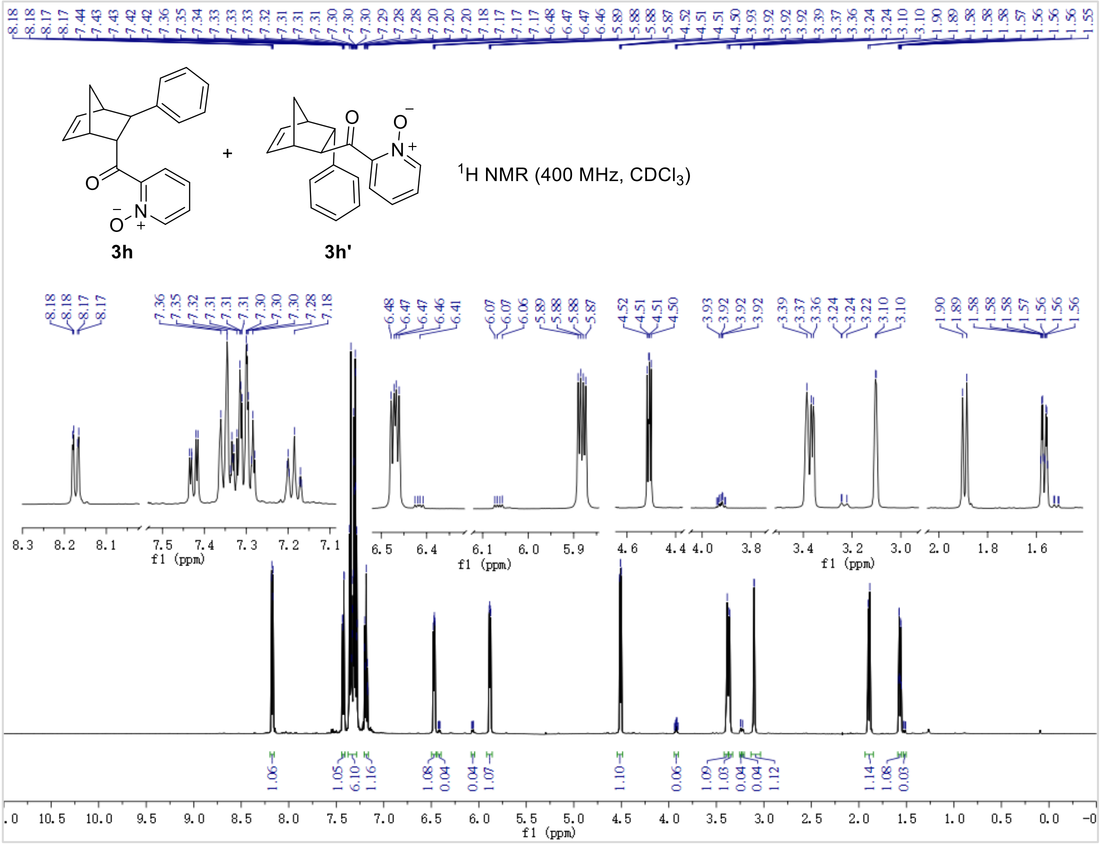

^{1}H NMR of **3h+3h’** (400 MHz, CDCl$_3$, mixture of *endo*/*exo* products)

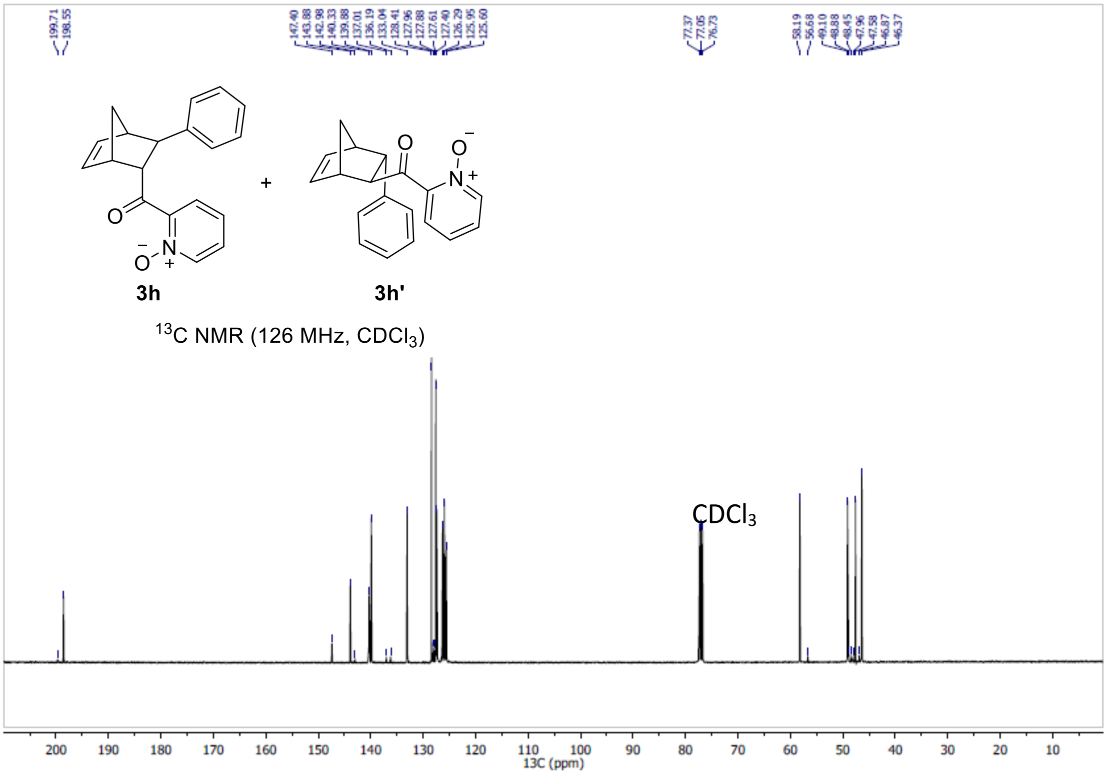

^{13}C NMR of **3h+3h’** (126 MHz, CDCl$_3$, mixture of *endo*/*exo* products)

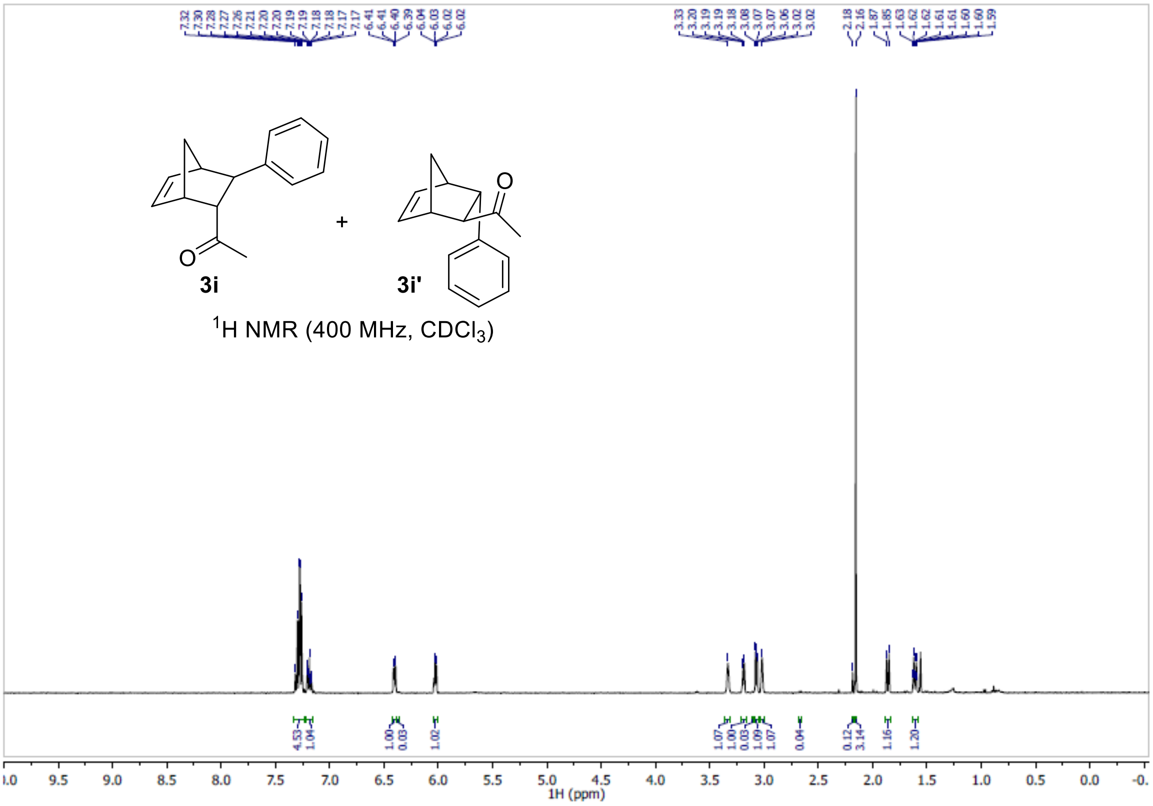

^{1}H NMR of **3i+3i'** (400 MHz, $CDCl_3$, mixture of *endo*/*exo* products)

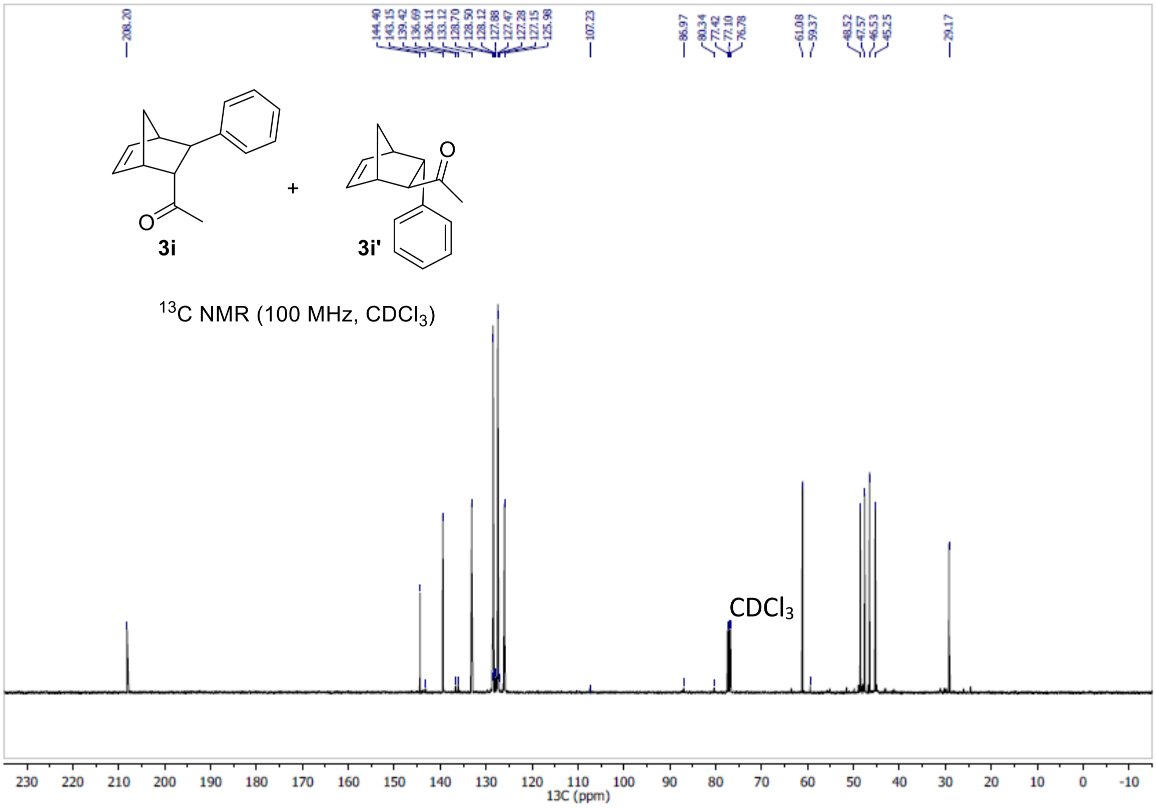

^{13}C NMR of **3i+3i'** (100 MHz, $CDCl_3$, mixture of *endo*/*exo* products)

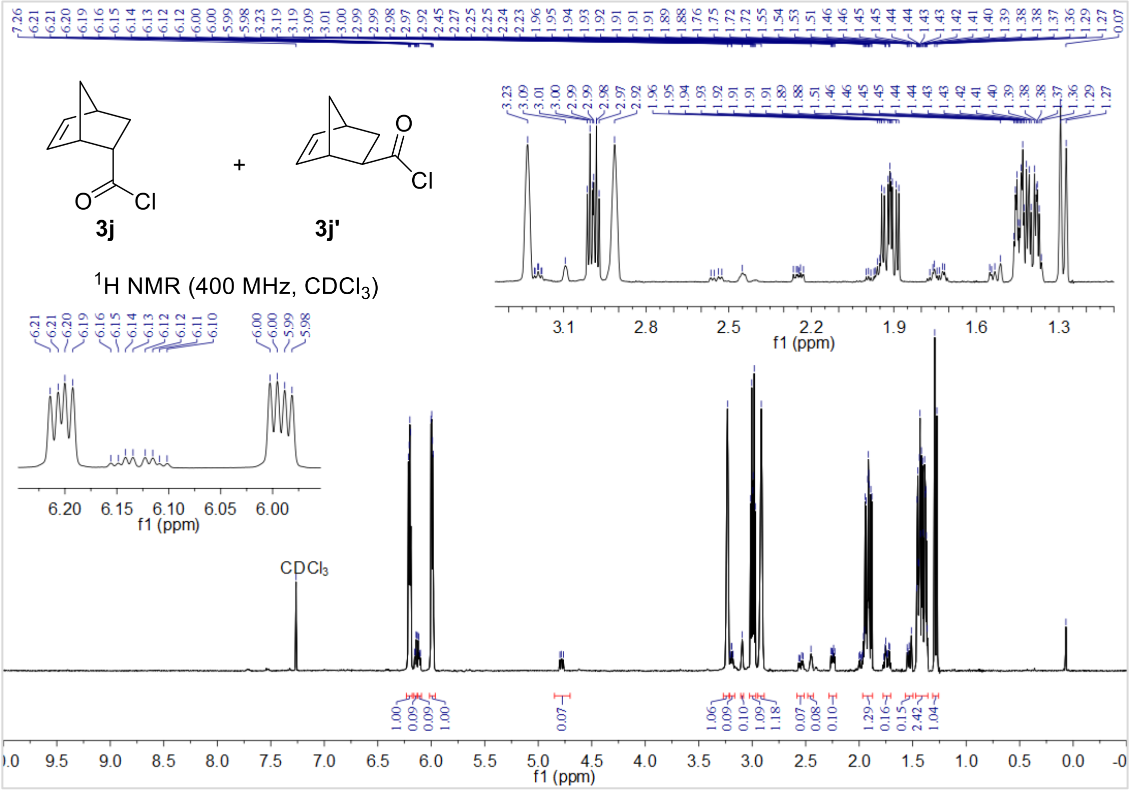

^{1}H NMR of **3j**+**3j'** (400 MHz, $CDCl_3$, mixture of *endo*/*exo* products)

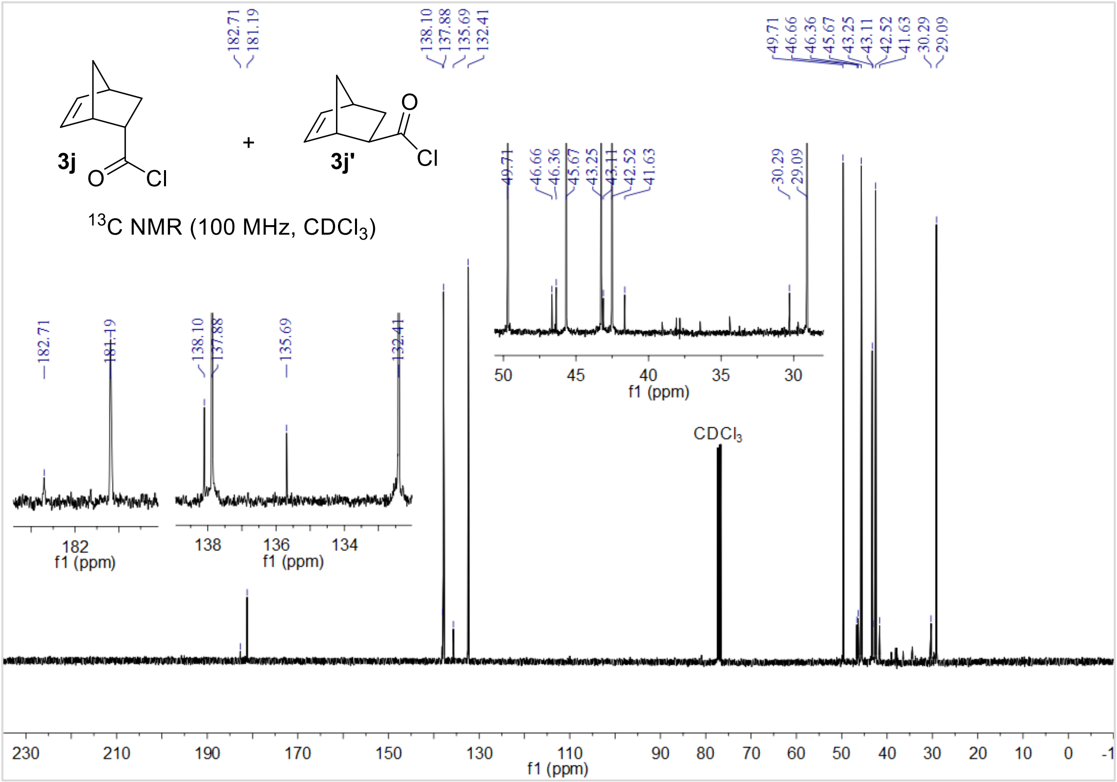

^{13}C NMR of **3j**+**3j'** (100 MHz, $CDCl_3$, mixture of *endo*/*exo* products)

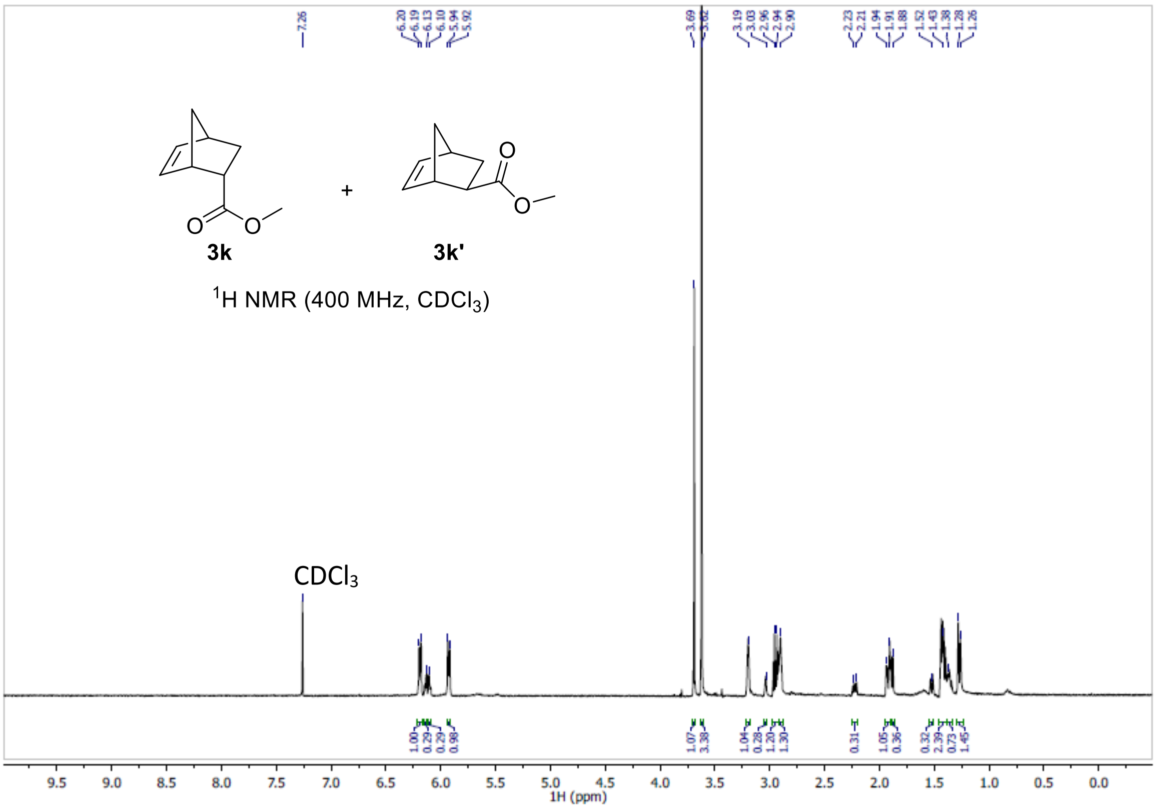

^{1}H NMR of **3k+3k'** (400 MHz, $CDCl_3$, mixture of *endo*/*exo* products)

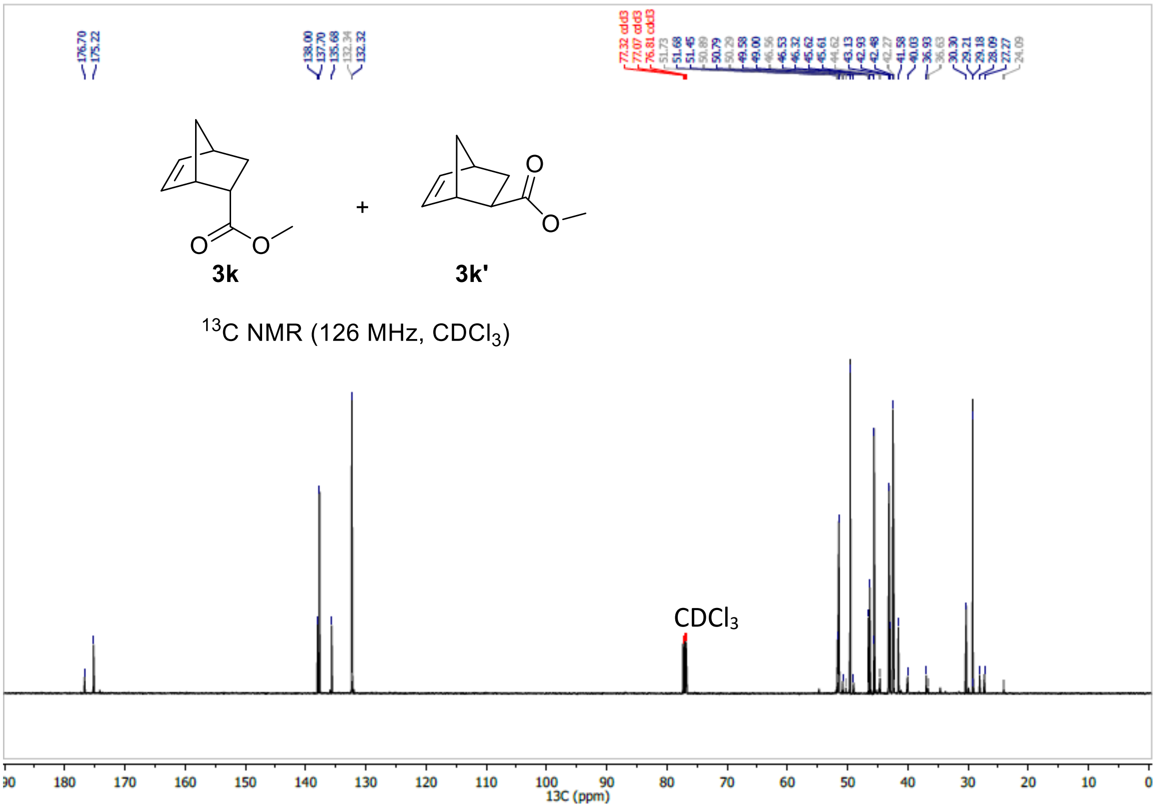

^{13}C NMR of **3k+3k'** (126 MHz, $CDCl_3$, mixture of *endo*/*exo* products)

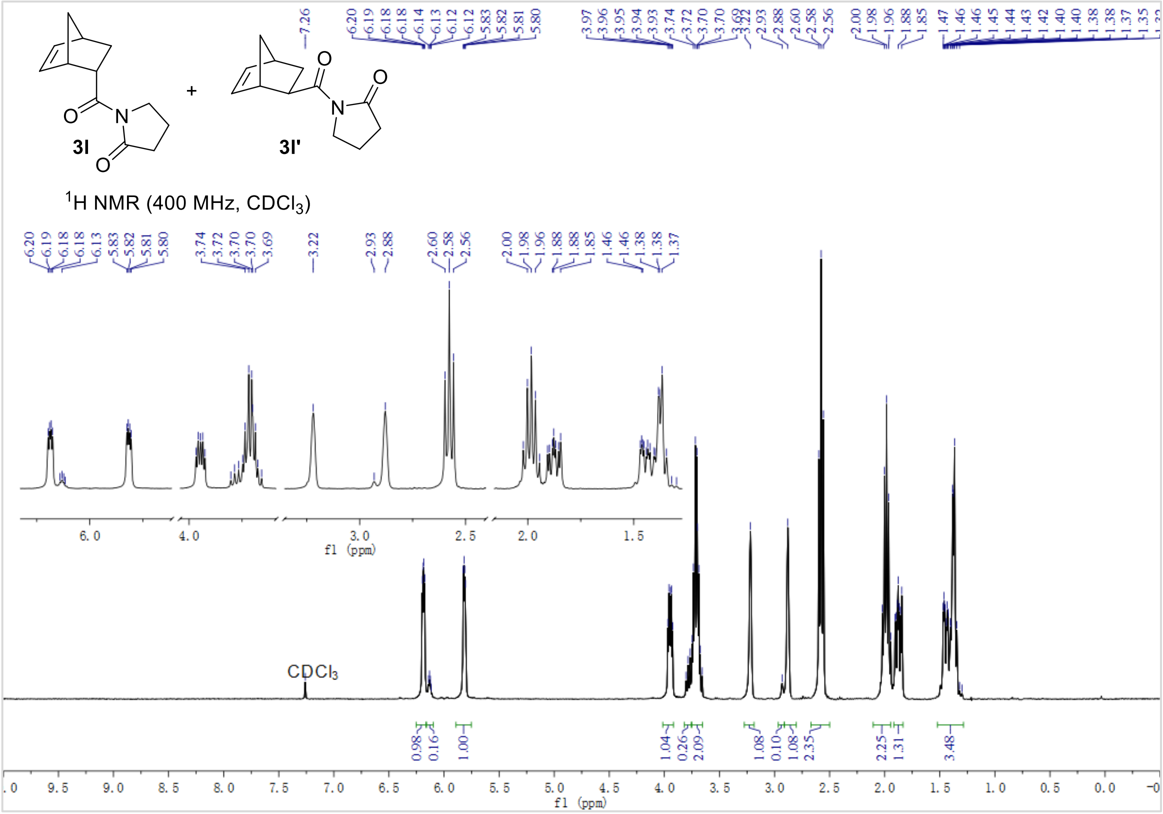

^{1}H NMR of **3l+3l'** (400 MHz, $CDCl_3$, mixture of *endo*/*exo* products)

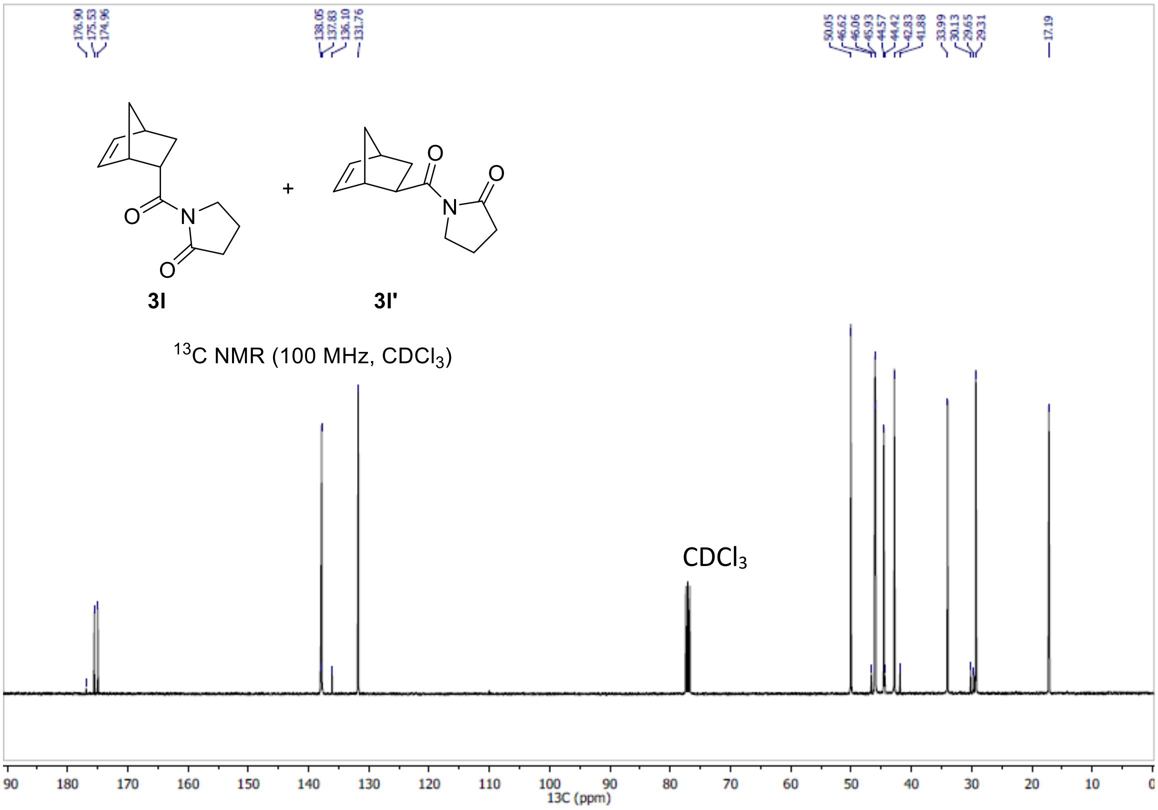

^{13}C NMR of **3l+3l'** (100 MHz, $CDCl_3$, mixture of *endo*/*exo* products)

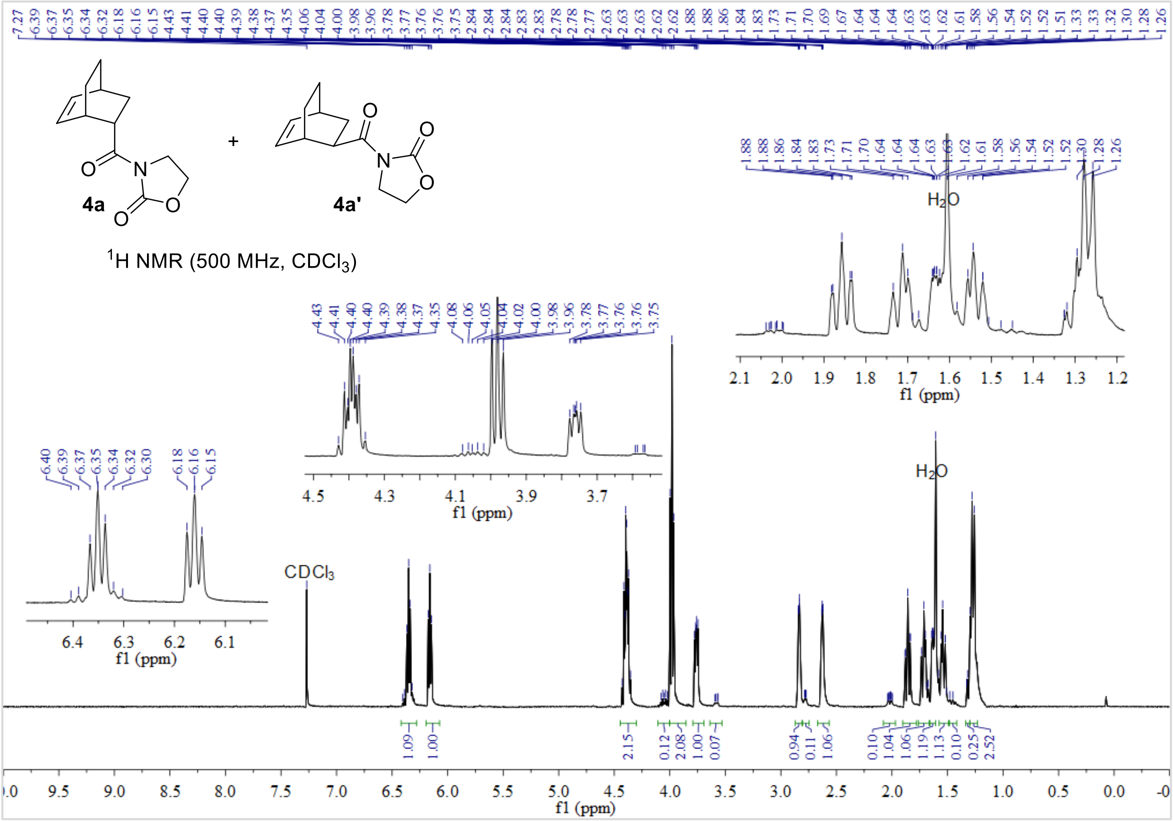

^{1}H NMR of **4a+4a'** (500 MHz, CDCl$_3$, mixture of *endo*/*exo* products)

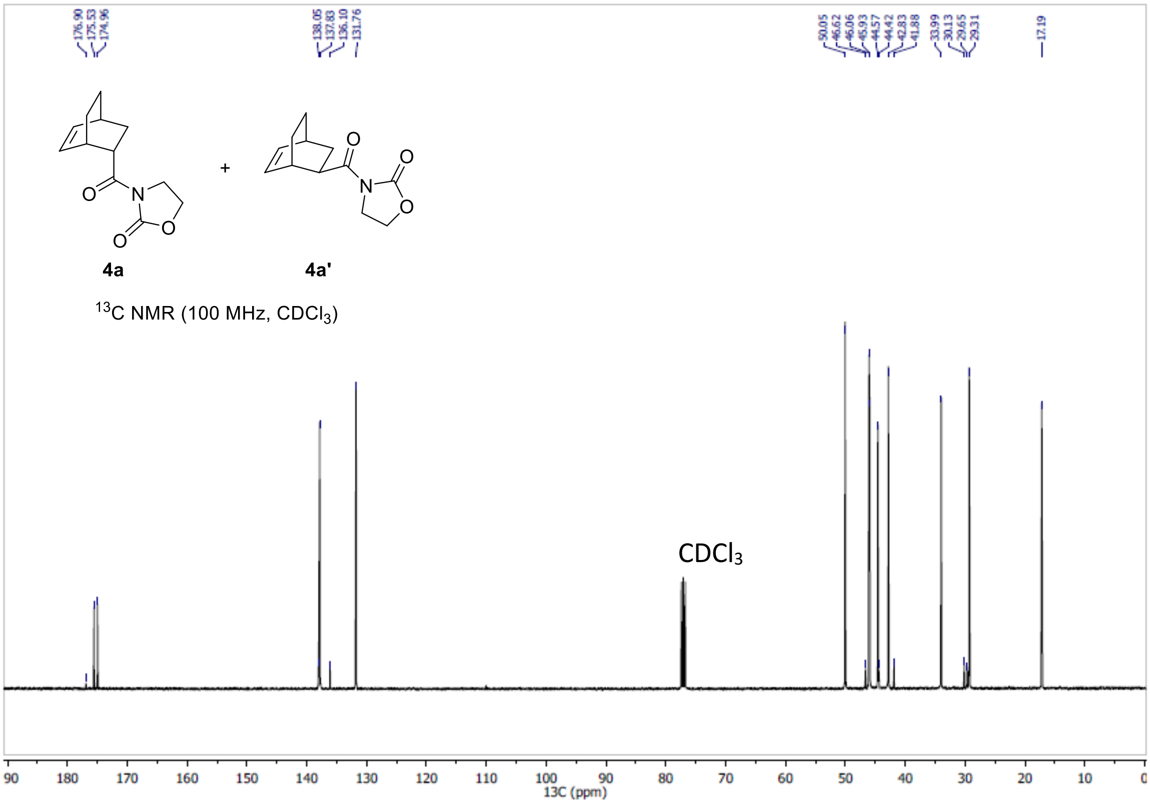

^{13}C NMR of **4a+4a'** (100 MHz, CDCl$_3$, mixture of *endo*/*exo* products)

5. References

1 Pinto, R. M. A.; Salvador, J. A. R.; Le Roux, C. *Catal. Commun.* 2008, **9**, 465-469.

2 Grommet, A. B.; Bolliger, J. L.; Browne, C.; Nitschke, J. R. *Angew. Chem. Int. Ed.* 2015, **54**, 15100-15104.

3 Matson, E. M.; Bertke, J. A.; Fout, A. R. *Inorg. Chem.* 2014, **53**, 4450-4458.

4 Blakesley, D. W.; Payne, S. C.; Hagen, K. S. *Inorg. Chem.* 2000**, 39**, 1979-1989.

5 Kascatan-Nebioglu, A.; Panzner, M. J.; Garrison, J. C.; Tessier, C. A.; Youngs, W. J., *Organometallics* 2004, **23**, 1928-1931.
